# Identification of m6A-Related lncRNAs Associated With Prognoses and Immune Responses in Acute Myeloid Leukemia

**DOI:** 10.3389/fcell.2021.770451

**Published:** 2021-11-16

**Authors:** Ding Li, Jiaming Liang, Cheng Cheng, Wenbin Guo, Shuolei Li, Wenping Song, Zhenguo Song, Yongtao Bai, Yongna Zhang, Xuan Wu, Wenzhou Zhang

**Affiliations:** ^1^ Department of Pharmacy, Affiliated Cancer Hospital of Zhengzhou University, Henan Cancer Hospital, Zhengzhou, China; ^2^ State Key Laboratory of Respiratory Disease, National Clinical Research Center for Respiratory Disease, The First Affiliated Hospital of Guangzhou Medical University, Guangzhou, China; ^3^ Department of Hematology, Affiliated Cancer Hospital of Zhengzhou University, Henan Cancer Hospital, Zhengzhou, China; ^4^ Department of Pathology, Pingtan Comprehensive Experimental Area Hospital, Fuzhou, China; ^5^ Department of Internal Medicine, Affiliated Cancer Hospital of Zhengzhou University, Henan Cancer Hospital, Zhengzhou, China

**Keywords:** M6A, long noncoding RNA, prognostic signature, immunotherapy response, acute myeloid leukemia

## Abstract

**Background:** Acute myeloid leukemia (AML) remains the most common type of hematopoietic malignancy in adults and has an unfavorable outcome. Herein, we aimed to construct an N6-methylandenosine (m6A)-related long noncoding RNAs (lncRNAs) signature to accurately predict the prognosis of patients with AML using the data downloaded from The Cancer Genome Atlas (TCGA) database.

**Methods:** The RNA-seq and clinical data were obtained from the TCGA AML cohort. First, Pearson correlation analysis was performed to identify the m6A-related lncRNAs. Next, univariate Cox regression analysis was used to determine the candidate lncRNAs with prognostic value. Then, feature selection was carried out by Least absolute shrinkage and selection operator (LASSO) analysis, and seven eligible m6A-related lncRNAs were included to construct the prognostic risk signature. Kaplan–Meier and receiver operating characteristic (ROC) curve analyses were performed to evaluate the predictive capacity of the risk signature both in the training and testing datasets. A nomogram was used to predict 1-year, 2-year, and 3-year overall survival (OS) of AML patients. Next, the expression levels of lncRNAs in the signature were validated in AML samples by qRT-PCR. Functional enrichment analyses were carried out to identify probable biological processes and cellular pathways. The ceRNA network was developed to explore the downstream targets and mechanisms of m6A-related lncRNAs in AML.

**Results:** Seven m6A-related lncRNAs were identified as a prognostic signature. The low-risk group hold significantly prolonged OS. The nomogram showed excellent accuracy of the signature for predicting 1-year, 2-year and 3-year OS (AUC = 0.769, 0.820, and 0.800, respectively). Moreover, the risk scores were significantly correlated with enrichment in cancer hallmark- and malignancy-related pathways and immunotherapy response in AML patients.

**Conclusion:** We developed and validated a novel risk signature with m6A-related lncRNAs which could predict prognosis accurately and reflect the immunotherapy response in AML patients.

## Introduction

Acute myeloid leukemia (AML) is one of the most aggressive hematological malignancies, with the highest incidence in adults (Short et al., 2018). Despite advancements in the biological understanding of AML, the standard treatment has not significantly improved over 40 years (Choi et al., 2020). The prognosis remains dismal, and most patients encounter disease recurrence or die of the disease several months after their initial remission. The long-term overall survival (OS) rate of young patients (<60 years) is less than 40%, and that of elderly patients (≥60 years) is only 15% (Koenig and Mims, 2020). Therefore, it is essential to identify novel molecules to accurately predict the prognosis and guide treatment in AML.

RNA methylation remains the frontier and focus of current epigenetic research, and N6-methyladenosine (m6A) is the main internal epigenetic modification in eukaryotic messenger RNAs (mRNAs) and noncoding RNAs (ncRNAs) (Deng et al., 2018). m6A affects the stability, alternative splicing, nuclear exit and translation efficiency of mRNA, and therefore plays important roles in various disease processes, including cancer (Cao et al., 2016; Wei et al., 2017; Dai et al., 2018). Recent research has revealed that the inactivation of m6A RNA methylases and demethylases in AML suppresses malignant cells through multiple m6A-dependent mechanisms. m6A RNA methylases METTL3/14 (Vu et al., 2017; Weng et al., 2018) and demethylases FTO/ALKBH5 (Huang et al., 2019; Shen et al., 2020), which are aberrantly expressed in specific leukemia subtypes, play a critical role in leukemogenesis by regulating specific gene targets and signaling pathways. Likewise, the m6A reader YTHDF2, which is highly expressed across AML subgroups, has been found to promote leukemogenesis by inhibiting gene targets through a YTHDF2-mediated mRNA decay mechanism (Paris et al., 2019). Therefore, m6A mRNA modification has promising therapeutic and prognostic potential in AML.

Long noncoding RNAs (lncRNAs), which possess no protein-coding capacity, are defined as transcripts longer than 200 nt and regarded as “noise” in genome transcription (Gibb et al., 2011). Previous studies have revealed that lncRNAs play essential roles in many important regulatory processes, such as transcription regulation, genome imprinting, chromatin modification, and nuclear transport, which have attracted widespread attention (Groff et al., 2016; Long et al., 2017). Some lncRNAs, including CASC15 (Fernando et al., 2017), UCA1 (Li et al., 2020), H19 (Zhang et al., 2018), HOTAIRM1 (Chen et al., 2020), LINC00152 (Cui et al., 2021), have been identified to be associated with the recurring mutations, clinical features and prognosis of AML. Accumulating studies have indicated that aberrant expression of certain specific lncRNAs in tumor cells can be used as a diagnostic marker or a potential drug target. Additionally, lncRNAs can easily be detected in serum and saliva, urine, blood, or tissue biopsy, which makes them attractive for clinical diagnosis and prognostic prediction (Chandra Gupta and Nandan Tripathi, 2017).

Extensive studies have showed that some m6A modifications could be directly or indirectly regulated by lncRNAs. The interaction of m6A modification and lncRNAs plays crucial roles in tumor progression, metastasis, response to immune, drug resistance, and offers new insights for early diagnosis and new treatment strategies of cancer (Chen et al., 2020). However, specific roles of the m6A-related lncRNAs in AML remains to be elucidated. Therefore, understanding how m6A-related lncRNAs work may enable to identify potential molecules as therapeutic targets. In this study, we first constructed a prognostic risk signature with seven m6A-related lncRNAs and further validated the reliability and sensitivity of the signature. Moreover, we also explored the correlation of the risk score and tumor microenvironment. Finally, we built a ceRNA and PPI networks in order to further study the potential mechanisms of m6A-related lncRNAs in AML.

## Materials and Methods

### Data Collection

We obtained the RNA-seq data and relevant clinical profiles of AML patients from The Cancer Genome Atlas (TCGA) database. After screening, cases with missing clinical data and/or OS ≤ 30 days were excluded from the study, and a total of 144 AML cases were included in the analysis. We subsequently transformed the probe IDs of each AML cohort into gene symbols based on the annotation files. The clinical characteristics for AML cases were summarized in Supplementary Table S1.

### Correlation Analysis

We retrieved 21 m6A gene expression matrixes from the TCGA AML cohort, including expression data on methyltransferases, binding proteins, and demethylases. The 21 m6A genes included erasers (ALKBH5 and FTO), writers (ZC3H13, KIA1429, RBM15, RBM15B, METTL3, METTL14, METTL16 and WTAP), and readers (RBMX, HNRNPA2B1, HNRNPC, IGF2BP1, IGF2BP2, IGF2BP3, YTHDC1, YTHDC2, YTHDF1, YTHDF2 and YTHDF3). 11,904 lncRNA expression data were extracted from the TCGA AML cohort. Subsequently, Pearson correlation analysis was applied to investigate the relationship between m6A genes and lncRNA expression to determine the m6A-related lncRNAs with a correlation coefficient >0.3 and *p* < 0.05.

### Generation of a Prognostic Signature Using LASSO Regularization

To establish a reliable signature, the 144 cases were randomly divided into training (100 cases) and testing datasets (44 cases). The log-rank test was performed to extract the m6A-related lncRNAs that were closely associated with survival time for development of the prognostic signature. Subsequently, least absolute shrinkage and selection operator (LASSO) was performed to exclude the candidate lncRNAs significantly associated with each other to restrict overfitting with the R package “glmnet.” Then, we identified seven m6A-related lncRNAs to build the signature through multivariate Cox proportional hazards regression analysis. The risk scores were calculated using the following formula based on the included m6A-related lncRNAs. Based on the median risk score, the samples were classified to high- and low-risk groups.

$$\begin{array}{c} Risk\;Score=0.1906265*\mathrm{USP}30-\mathrm{AS}1+0.10995083*\mathrm{AC}114271.2\\ +0.0704641*\mathrm{AF}064858.8+0.02511635*\mathrm{RP}11-22L13.1\\ +\left(-0.129286\right)*\mathrm{MIR}181A1\mathrm{HG}+\left(-0.0512868\right)*\mathrm{RP}11\\ -544A12.4+\left(-0.0383528\right)*\mathrm{MIR}133A1\mathrm{HG} \end{array}$$

### Survival Analysis

Kaplan–Meier curves analysis and log-rank tests were used to compare the discrepancy of OS between the predicted high-risk and low-risk groups. *p* ≤ 0.05 was defined as statistical difference. All survival analyses and log-rank tests were carried out using the R package survival, while the R package “surviminer” was used to plot the Kaplan–Meier curve.

### Construction and Evaluation of the Nomogram

R package “rms” was performed to construct a nomogram based on clinical stage, T stage and the risk score using the TCGA AML cohort. To evaluate the utility of the nomogram, the R package “ROC survival” was used to construct ROCs for the prediction of the 1-, 2- and 3-year OS by the nomogram. The R package “ggDCA” was used to construct a decision analysis curve to evaluate the clinical utility. Finally, package “rms” was used to establish a calibration curve to assess the precision for prediction of 1-, 2- and 3-year OS.

### Functional Enrichment Analysis

Gene Ontology (GO) and Kyoto Encyclopedia of Genes and Genomes (KEGG) enrichment analyses of the differentially expressed genes (DEGs) in the two risk groups were performed by “cluster Pofolier” package. (Yu et al., 2012). Further, Gene Set Enrichment Analysis (GSEA) was used to analyze the biological functions and pathways associated with the high and low-risk groups. FDR <0.05 and *p* < 0.05 were regarded statistically significant.

### CeRNA Network

MiRcode was used to predict the miRNAs that interact with m6A-related lncRNAs. A total of 25 pairs of interactions between 9 lncRNAs and 10 miRNAs were identified by the miRcode database. A total of 271 mRNAs interacting with the miRNAs were identified to construct a PPI network through TargetScan, miRDB, and miR TarBase. The ceRNA network was visualized using Cytoscape software. The Cyto Hubba plugin was performed to obtain hub genes from the PPI network. GO and KEGG enrichment analyses were conducted to identify biological processes and potential signaling pathways.

### Sample Collection

We totally collected 21 bone marrow samples, including 14 AML samples (derived from 7 AML patients at the time of first diagnosis and at first relapse) and 7 healthy controls in the Hematological Department of The Affiliated Cancer Hospital of Zhengzhou University. This research was approved by the Medical Ethics Committee of The Affiliated Cancer Hospital of Zhengzhou University (approval no. 2020239). Informed consent and approval were provided by all participants.

### qRT-PCR Analysis

Total RNA was isolated from 21 patients’ samples. cDNA synthesis was conducted with a reverse transcription kit (TransGen Biotech, #AU311-02). Then, real-time PCR was performed on the ABI 7500 Fast System (Applied Biosystems, United States) with TB Green Premix Ex Taq (Takara Bio, #RR420A). Relative expression of lncRNAs were normalized to GAPDH and calculated by 2-ΔΔCt method. Primers sequences are listed in Supplementary Table S2.

### Statistical Analysis

All statistical data were analyzed using R version 4.0.2. Pearson’s correlation analyses were applied to evaluate the correlation between the risk score and specific gene expression. Kaplan–Meier curves and log-rank tests were conducted for survival analysis. Univariate and multivariate Cox regression were applied to assess the prognostic independence. The reliability and sensitivity of the signature were evaluated using ROC curve analysis. Student’s *t*-test was used to determine significance between two groups. *p* < 0.05 was defined as statistical significance.

## Results

### Construction of the m6A-Related lncRNAs Prognostic Signature for AML Patients

**FIGURE 1:**
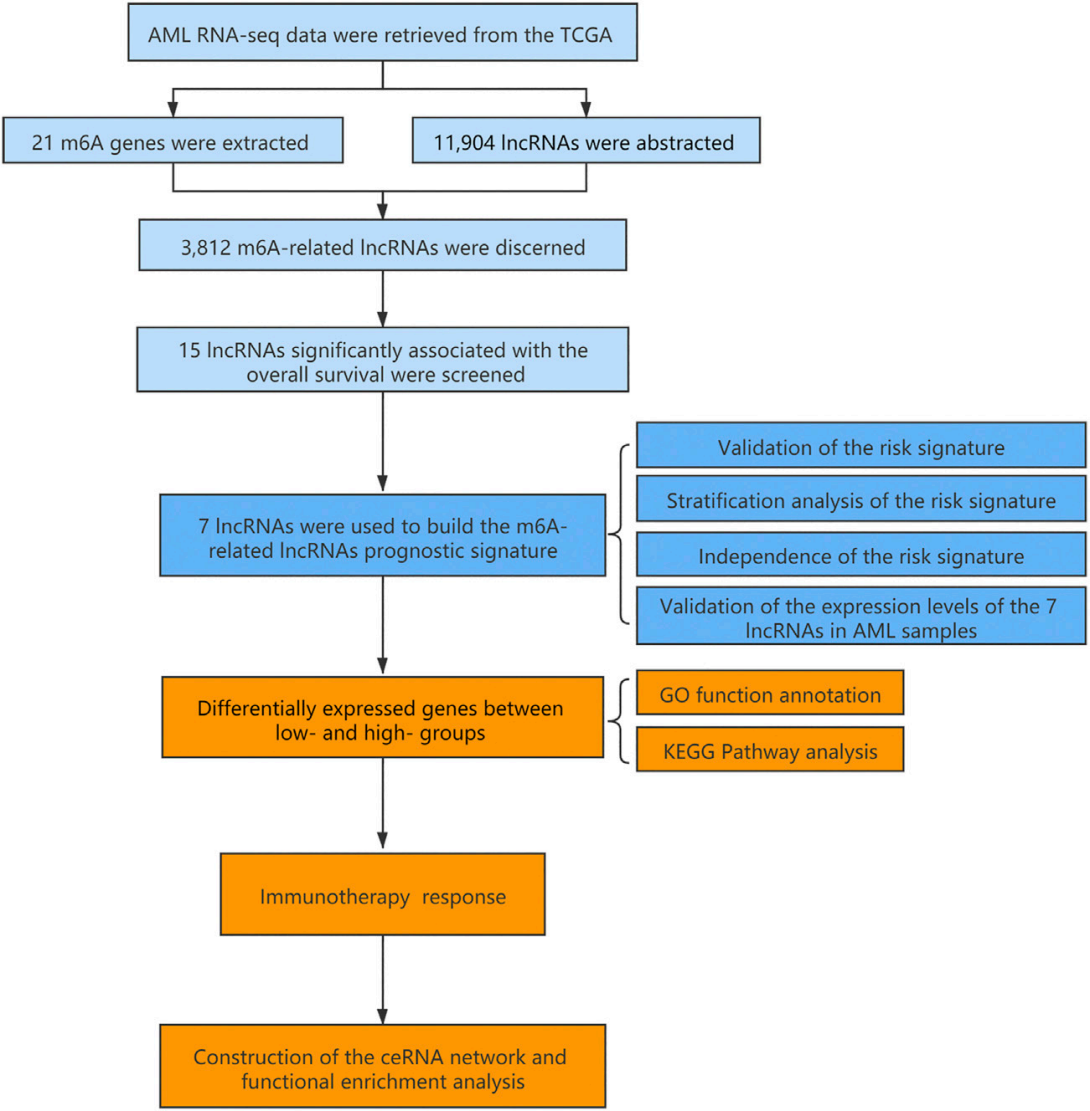
Flow chart of the design in the study.

**FIGURE 2:**
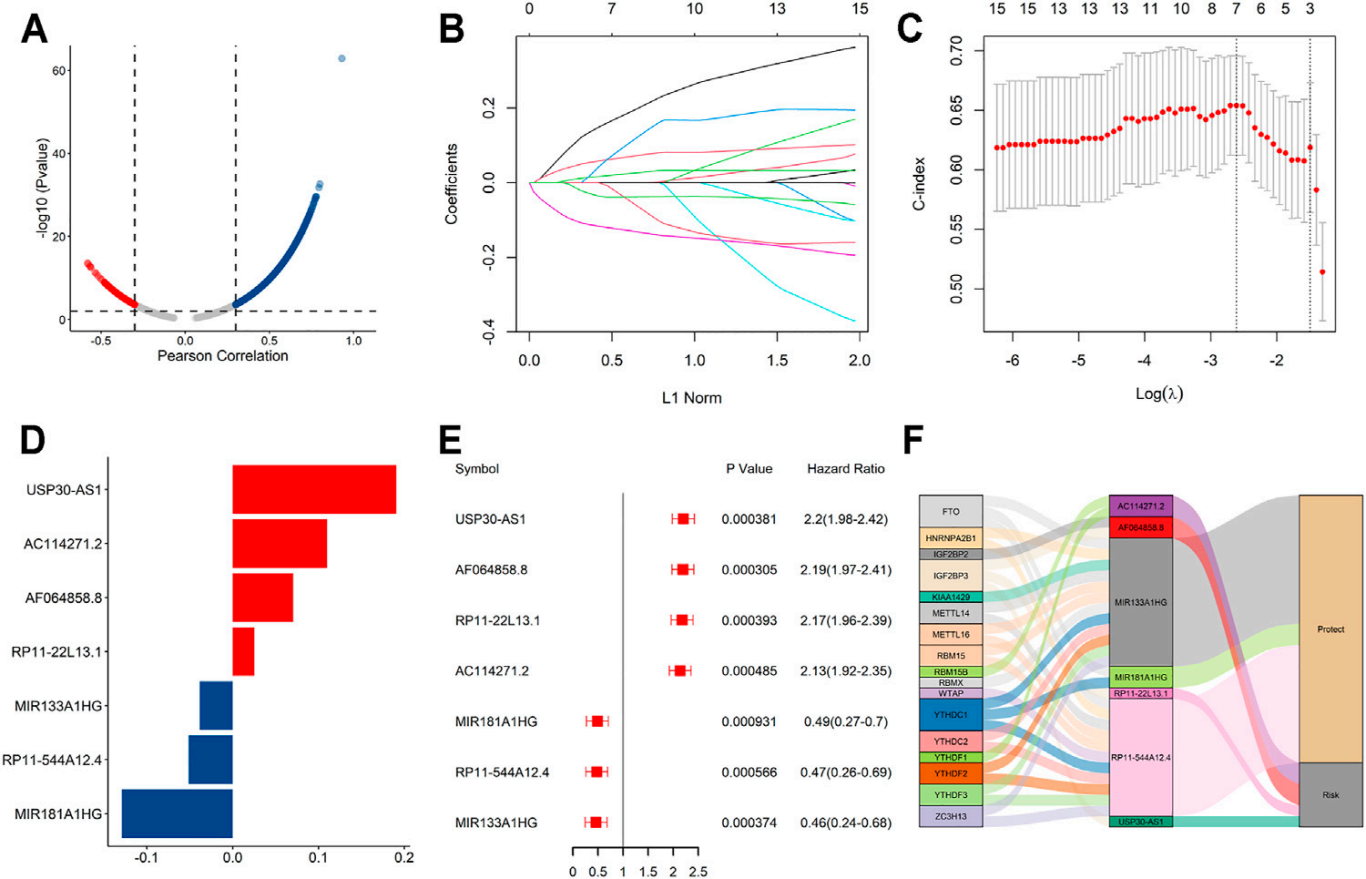
A seven m6A-related lncRNA prognostic signature was constructed for AML patients. **(A)** Pearson correlation analysis between m6A-related genes and lncRNA expression in AML samples. **(B,C)** LASSO coefficient values and vertical dashed lines were calculated at the best log(lambda) value. **(D)** LASSO coefficient profiles of the prognosis-related lncRNAs. **(E)** Forest plot of the univariate Cox regression analysis for the 7 prognosis-related lncRNAs. **(F)** The Sankey diagram shows the correlation between m6A genes, m6A-related lncRNAs and the risk type.

The flowchart summarized the construction and subsequent analysis of the risk signature (Figure 1). The expression profiles of 21 well known m6A genes and 11,904 lncRNAs were extracted from TCGA AML cohort. The m6A-related lncRNAs were defined as ones which were associated with one or more of these m6A genes (|Pearson R| > 0.3 and *p* < 0.05), and a total of 3,812 lncRNAs were included (Figure 2A). Next, 15 m6A-related lncRNAs significantly related to the OS of AML patients were screened out through univariate Cox regression analysis (*p* < 0.001). Then, feature selection was performed using LASSO analysis, and 7 m6A-related lncRNAs were ultimately identified to develop the prognostic risk signature (Figures 2B–D). Among them, USP30-AS1, AC114271.2, AF064858.8 and RP11-22L13.1 are detrimental factors with a hazard ratio (HR) > 1, whereas MIR181A1HG, RP11-544A12.4 and MIR133A1HG are protective factors with a HR < 1 (Figure 2E). Sankey diagram shows the relationship between the lncRNAs and m6A genes and the risk types (Figure 2F).

### Validation of the Signature Performance in the TCGA Dataset

**FIGURE 3:**
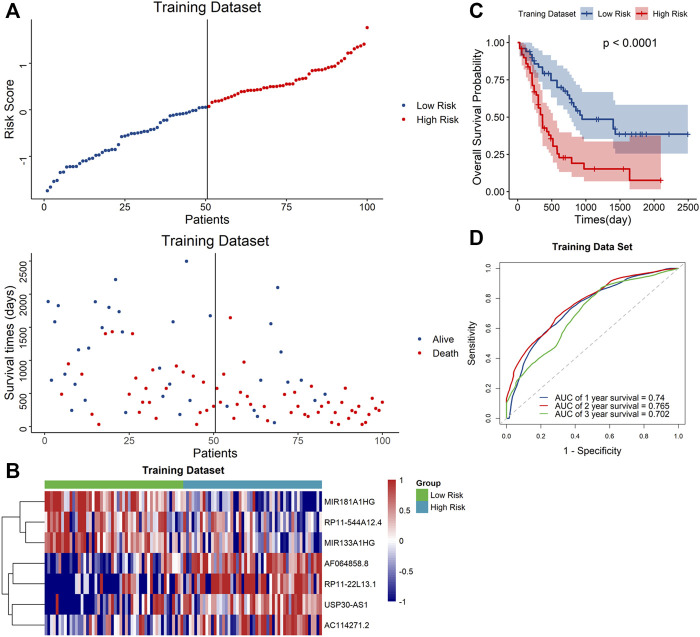
Validation of the signature in the training dataset **(A)**. Distribution of the risk score (top) and survival time of each patient (down). **(B)** Expression of the seven lncRNAs in the two risk groups. **(C)** Kaplan–Meier curve shows results of survival analysis for the signature in training dataset. **(D)** ROC curves analysis and the AUC values of 1-, 2-, and 3-year survival prediction in training dataset.

Based on the median risk score, the samples were classified to high- and low-risk groups. The scatter plot showed that mortality increased with a higher risk score (Figure 3A). Furthermore, the heatmap showed that the expression level of AF064858.8, RP11-22L13.1, USP30-AS1 and AC114271.2 were higher in the high-risk group, whereas MIR181A1HG, RP11-544A12.4 and MIR133A1HG were at lower expressed levels (Figure 3B). Moreover, Kaplan–Meier curve analyses indicated that the low-risk group had prolonged OS (Figure 3C). In addition, the AUC values for the 1-year, 2-year, and 3-year OS were 0.74, 0.765 and 0.702, respectively (Figure 3D), suggesting the high predictive capacity of the signature in the training dataset.

**FIGURE 4:**
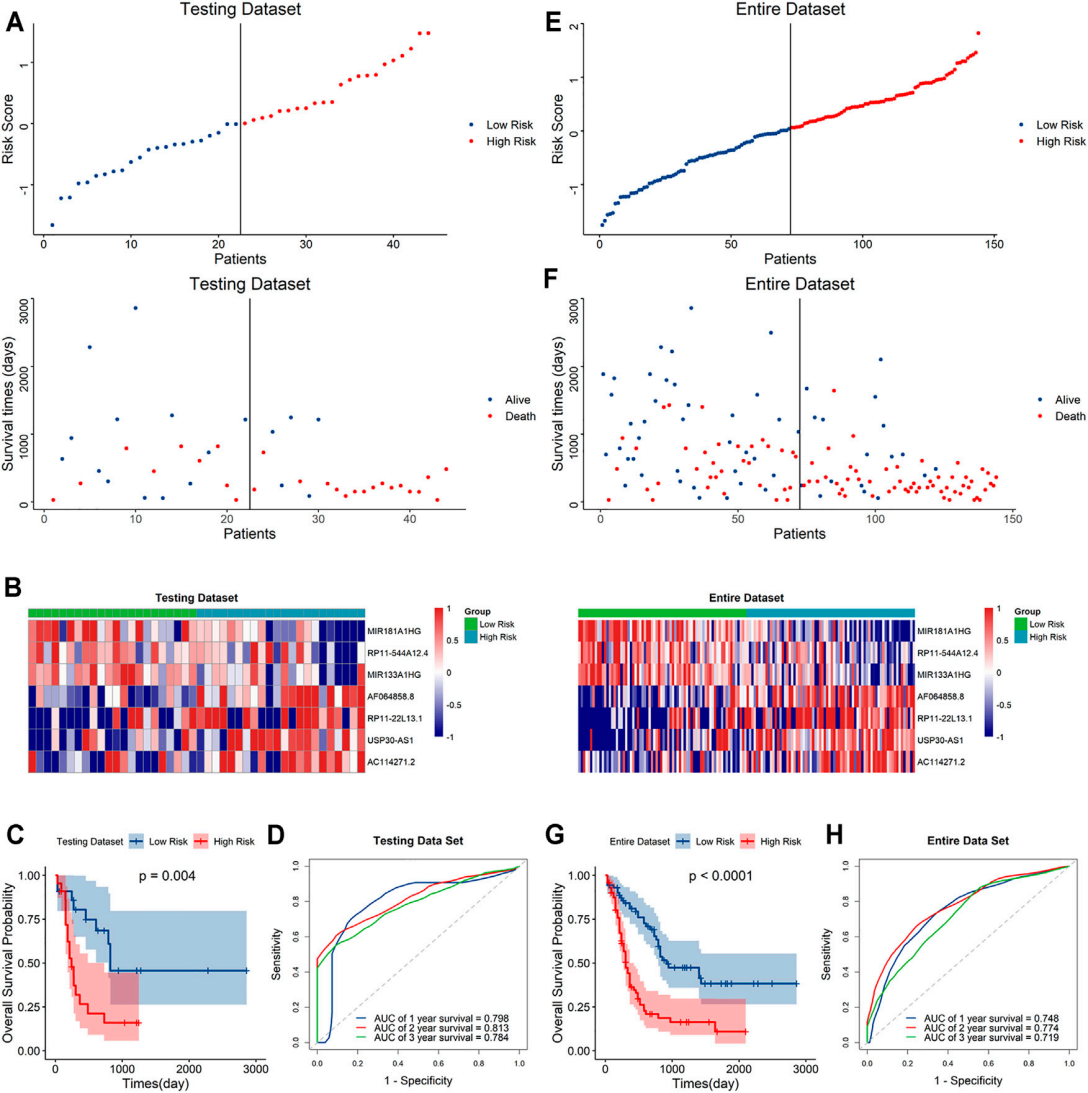
Evaluation of the signature in the testing and whole datasets **(A,E)**. Distribution of the risk score (top) and survival time of each patient (bottom) in the testing dataset **(A)** and whole dataset **(E)**. **(B,F)** Expression of the seven lncRNAs in the high- and low-risk groups in the testing dataset **(B)** and whole dataset **(F)**. **(C,G)** Kaplan–Meier curve analysis of the signature in the testing dataset **(C)** and whole datasets **(G)**. **(D,H)** ROC curves and their AUC values showed 1-, 2-, and 3-year predictions in the testing set **(D)** and whole dataset **(H)**.

To further validate the predictive capacity of the signature, we used the same algorithm to calculate the risk scores both in the testing dataset and the overall dataset. The risk score distribution plot, scatter plot, and heatmaps were consistent with those in the training dataset. Furthermore, Kaplan–Meier curve analyses showed the consistent results in the testing dataset and overall dataset (*p* < 0.001). In addition, the time-ROC curves and their AUC values also displayed good performance for predicting prognosis. The AUCs for 1-year, 2-year and 3-year OS in the testing dataset were 0.798, 0.813 and 0.784, and in the overall dataset, the AUCs were 0.748, 0.778 and 0.719, respectively (Figure 4). In summary, the high-risk score based on the signature could indicate a poor prognosis, accurately.

### Stratification Analyses of the Signature With Clinicopathological Characteristics

**FIGURE 5:**
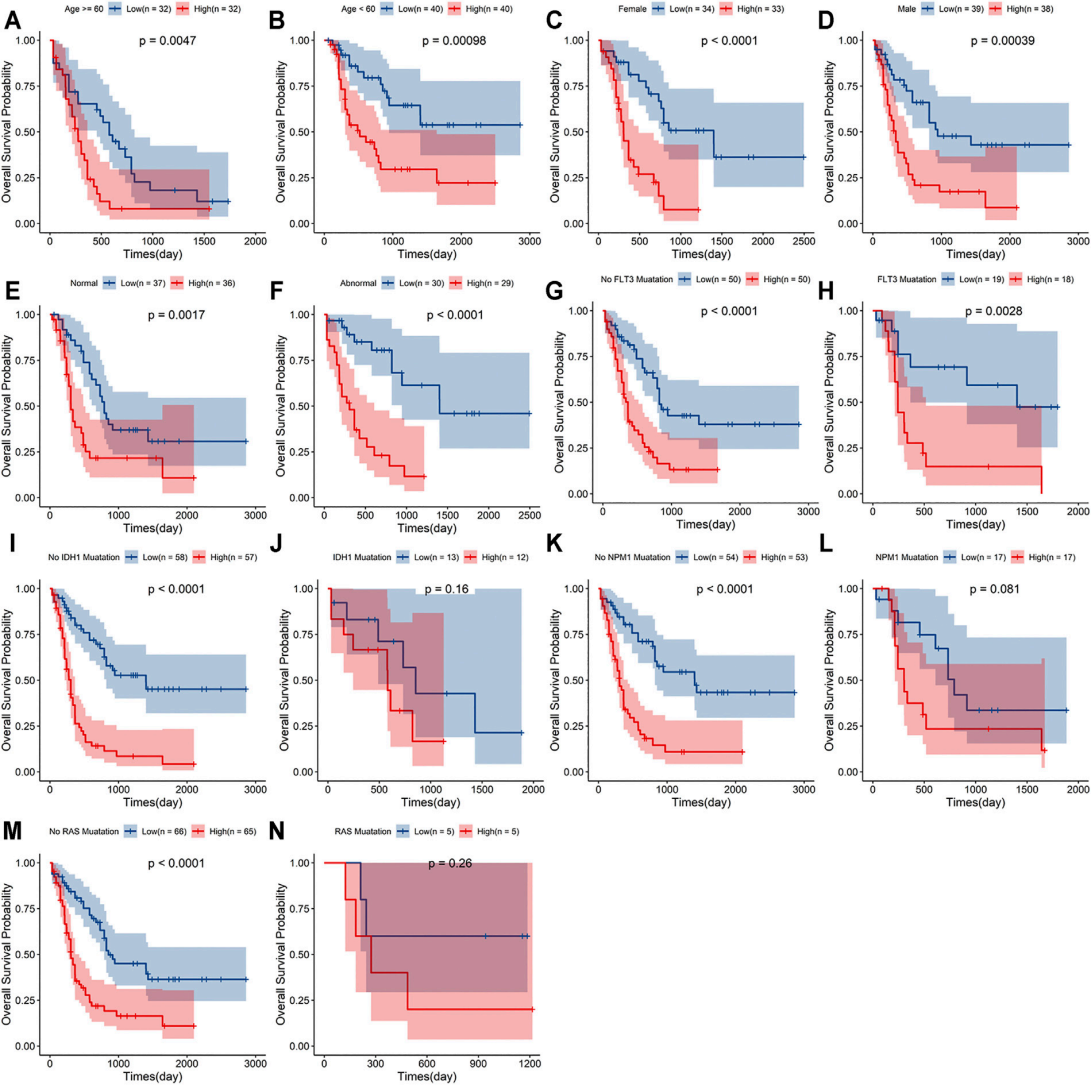
Stratification analyses of the signature based on prognosis-related clinicopathological characteristics. The survival outcomes of the patients stratified according to age **(A,B)**, sex **(C,D)**, chromosomal abnormalities **(E,F)**, FLT3 mutations **(G,H)**, IDH1 mutations **(I,J)**, NPM1 mutations **(K,L)** and RAS mutations **(M,N)** in the two risk groups.

A stratified analysis was carried out according to the clinicopathological characteristics, including age (<60/≥60 years, Figures 5A,B), sex (female/male, Figures 5C,D), abnormalities (normal/abnormal, Figures 5E,F), chromosomal FLT3 mutations (no/yes, Figures 5G,H), IDH1 mutations (no/yes, Figures 5I,J), NPM1 mutations (no/yes, Figure 5K,L), and RAS mutations (no/yes, Figures 5M,N). Kaplan–Meier curve analyses revealed that high-risk group had worse survival outcome than low-risk group when stratified by the different clinical features, except for IDH1, NPM1 or RAS mutations.

### Independent Prognostic and Clinicopathological Correlation Analyses

**FIGURE 6:**
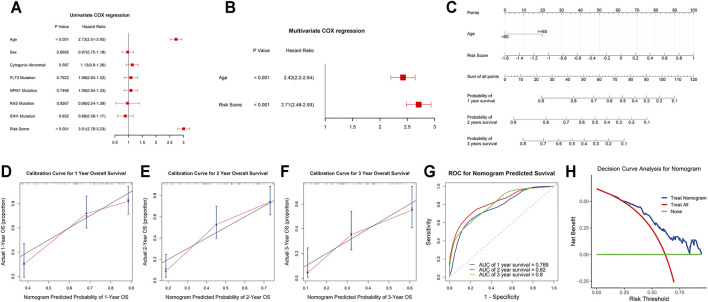
Independent prognostic and clinicopathological correlation analyses **(A,B)** Cox regression analysis of the clinical variables and risk scores. **(C)** Construction of a nomogram for 1-, 2-, and 3-year survival prediction along with risk score and age. **(D–F)** Calibration curves evaluated the consistency of nomogram. **(G)** Time-dependent ROC curves of the nomogram and their AUC values at 1-, 2-, and 3-year. **(H)** The decision curve analysis of the nomogram.

Univariate and stepwise multivariate Cox regression analyses were applied to explore whether the m6A-related lncRNAs signature and clinical characteristics, such as age, sex, cytogenic abnormalities, FLT3 mutation, IDH1 mutation, NPM1 mutation and RAS mutation, may serve as independent prognostic factors. Finally, age and risk score were selected as independent prognostic factors for the survival prediction (Figures 6A,B). Next, to quantitatively predict the survival probability of each case, a prognostic nomogram incorporating the clinical features and the signature was plotted (Figure 6C). Furthermore, calibration curves of 1-year, 2-year, and 3-year OS were plotted to confirm the predictive probability of the nomogram. The results showed that the predicted survival probability by the nomogram was consistent to the actual one (Figures 6D–F). Moreover, time-dependent ROC curves revealed that the nomogram showed remarkable accuracy to predict 1-, 2- and 3-year OS (AUC = 0.769, 0.82, and 0.8, respectively) (Figure 6G). In addition, the decision curve analysis of the LNM nomogram showed that even if the threshold probability of the patient is very small, the use of the LNM nomogram in predicting LNM brings more benefit than treating either all or no patients (Figure 6H). Above all, this signature displayed good performance in predicting the survival of AML.

### Validation of the Expression Levels of Seven m6A-Related lncRNAs in AML Samples

Besides, Kaplan-Meier curve analyses were used to evaluate the prognostic role of each gene from the signature, and the results showed that higher expression levels of USP30-AS1, AC114271.2, AF064858.8 and RP11-22L13.1, and lower expression levels of MIR181A1HG, RP11-544A12.4 and MIR133A1HG were associated with poorer survival outcomes (Supplementary Figure S1).

**FIGURE 7:**
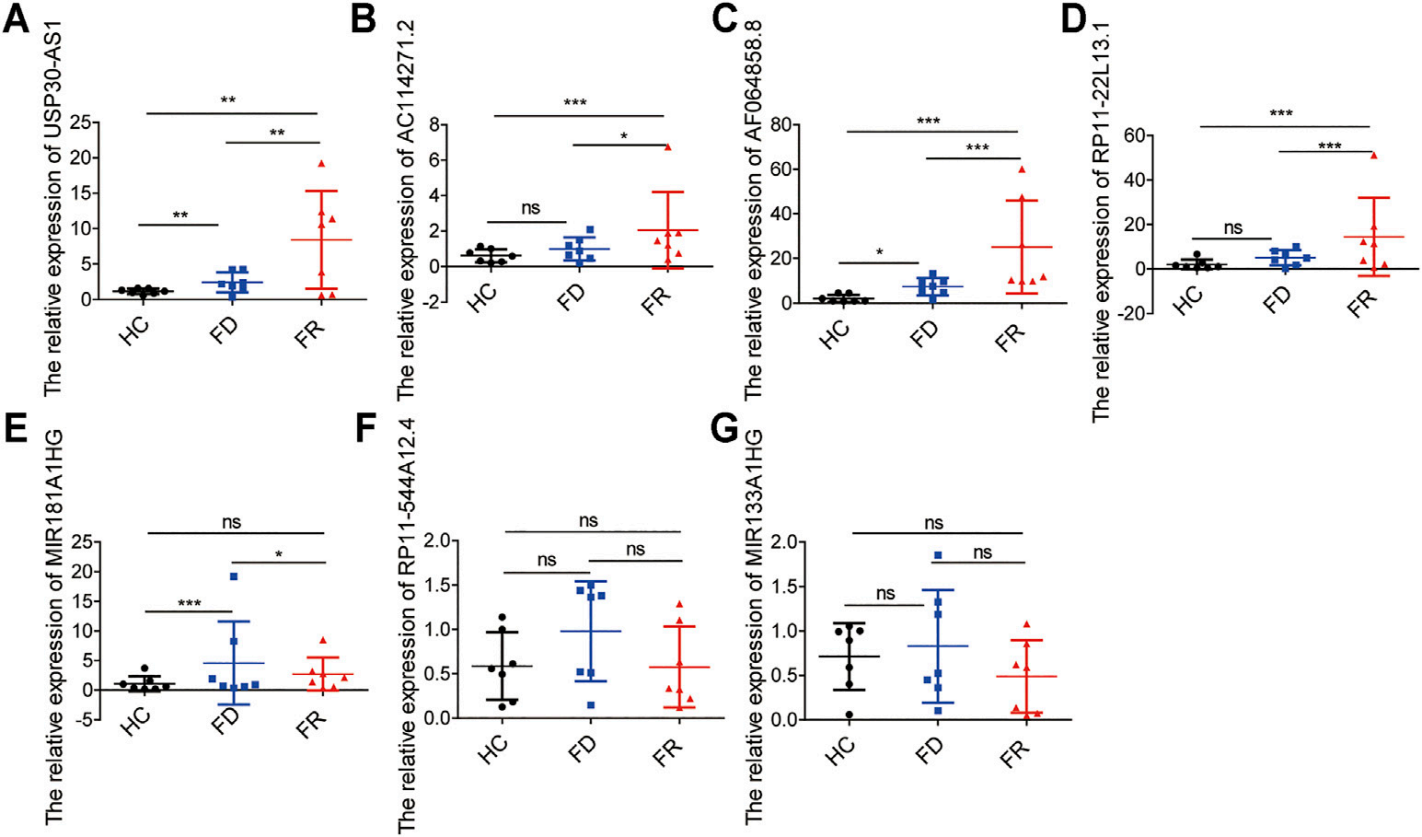
The expression levels of seven m6A-related lncRNAs in AML samples **(A–G)** the expression levels of seven lncRNAs in the signature detected by qRT-PCR, HC, Healthy Controls; FD, First Diagnosis; FR, First Relapse. *p < 0.05; **p < 0.01; ***p < 0.001.

To further demonstrate the feasibility of the prognostic signature and measure the potential in clinical practice, we performed qRT-PCR assays in our collected bone marrow samples. Our results showed that seven of the m6A-related lncRNAs could be easily detected both in AML patients and healthy controls. And, the expression levels of these lncRNAs were relatively higher in AML patients than healthy controls. It is worthy of note, compared with samples at first diagnosis, the detrimental factors of USP30-AS1, AC114271.2, AF064858.8 and RP11–22L13.1 were upregulated, and the protective factors of MIR181A1HG, RP11–544A12.4 and MIR133A1HG exhibited a decreased tendency in first relapsed AML patients (Figure 7). Generally speaking, the relapsed AML patients always encounter therapy resistance, resulting in poor survival outcomes, which consistent with our experimental validation in AML samples.

### Cellular Biological Effects Related to the Signature

**FIGURE 8:**
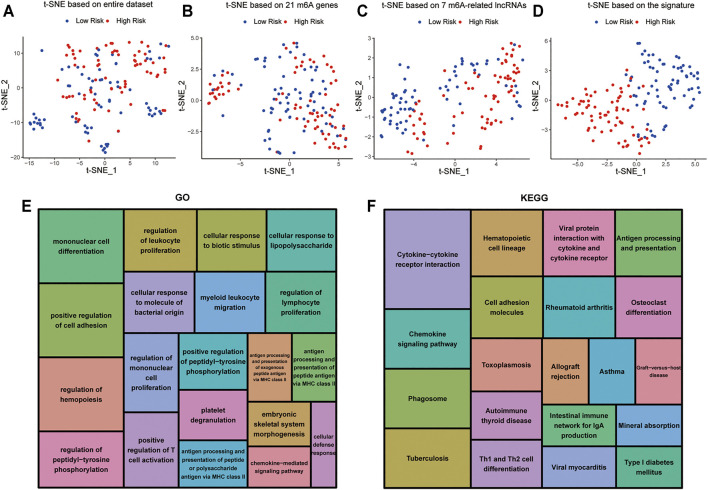
Cellular biological effects related to the signature **(A)** Based on whole gene expression profiles. **(B)** Based on 21 m6A genes. **(C)** Based on 7 m6A-related lncRNAs. **(D)** Based on the signature. **(E)** GO function annotation showed the top 20 enriched biological processes. **(F)** KEGG pathway analysis showed the top 20 enriched pathways.

t-distributed stochastic neighborhood embedding (t-SNE) was used to investigate the differences between the low-risk and high-risk groups. The results obtained based on entire genes, 21 m6A genes and 7 m6A-related lncRNAs showed that the distributions were relatively scattered (Figures 8A–C). However, the result obtained based on the signature showed that the two risk groups have different distributions, which suggested that the prognostic signature can easily distinguish the low-risk and high-risk groups (Figure 8D).

In view of the excellent predictive capacity of this signature, we further investigated the biological effect related to the molecular heterogeneity. We first identified 1,186 DEGs between the two risk groups. And the DEGs were primarily enriched in the following terms, including cellular defense response, mononuclear cell differentiation, chemokine-mediated signaling pathway and positive regulation of T cell activation (GO Biological Processes) (Figure 8E). Antigen processing and presentation, cytokine-cytokine receptor interaction, intestinal immune network for IgA production as well as Th1 and Th2 cell differentiation (KEGG Pathway) (Figure 8F). GSEA showed several tumor hallmarks were enriched in the high-risk group, such as interferon γ response, HEME metabolism, allograft rejection, interferon α response, complement, myogenesis, inflammatory response, KRAS signaling up, TNFα signaling via NF-κB and so on (Supplementary Figure S2). Above all, these evidences indicated that the signature is correlated with immune cell-related biological pathways and may be associated with the immune microenvironment in AML.

### Correlation of the Signature and Immunotherapy Response

**FIGURE 9:**
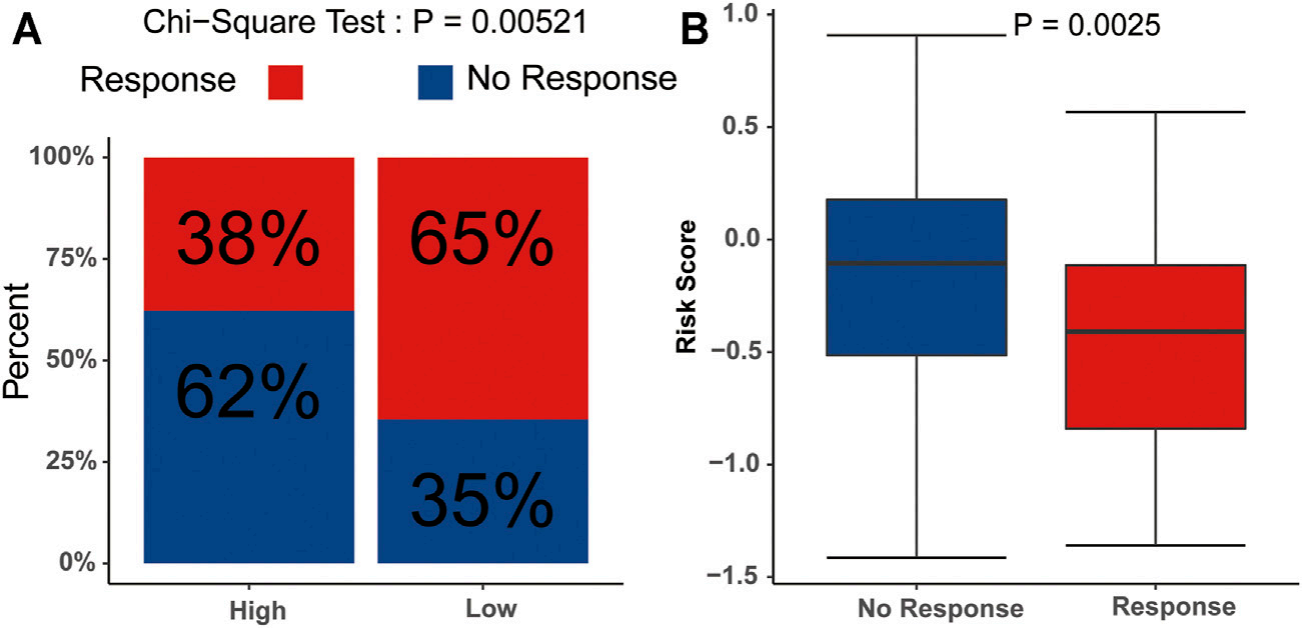
Potential relevance of the signature in immunotherapy response **(A)** Distribution of the immunotherapy responders in the low- and high-risk group. **(B)** Evaluation of the differences in risk scores between response group and non-response group.

We subsequently evaluated the immunotherapy response in patients with different risk scores. The analysis indicated that the low-risk group had a higher response rate than the high-risk group (*p*<0.05) (Figure 9A), and the risk scores in the no-response group were higher than those in the response group (Figure 9B). These results suggested that the signature might serve as a good tool to evaluate the immunotherapy response in AML.

### Construction of the ceRNA Network and Functional Enrichment Analysis

**FIGURE 10:**
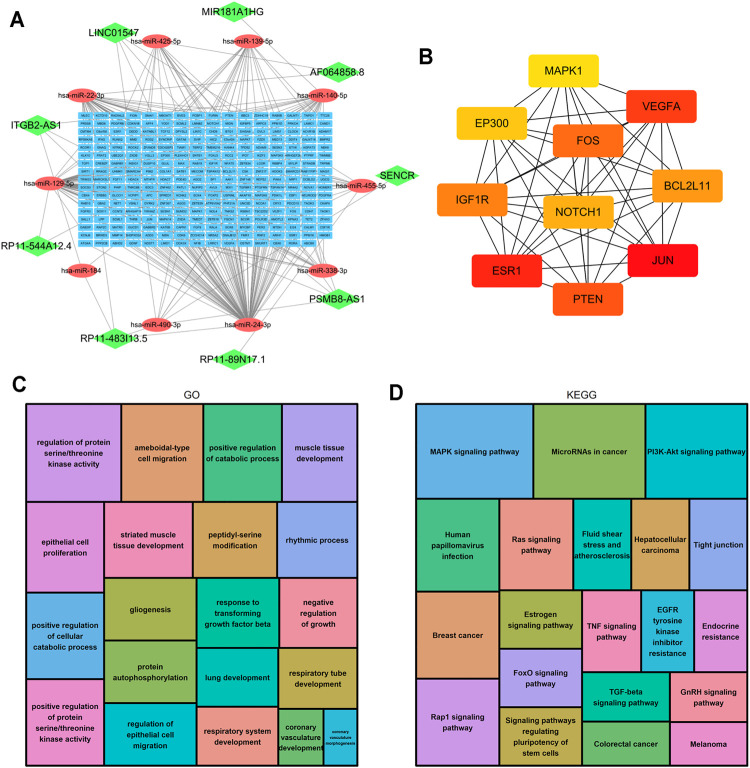
Construction of the ceRNA network and functional enrichment analysis. **(A)** The ceRNA network of lncRNAs (green diamonds), target miRNAs (red circles) and mRNAs (blue rectangles). **(B)** 10 hub genes were extracted from the PPI network. **(C)** GO functional annotation showed the top 20 enriched biological processes. **(D)** KEGG pathway analysis showed the top 20 enriched pathways.

LncRNAs can regulate the expression of downstream mRNAs by combining shared miRNAs as ceRNAs (Tay et al., 2014). Therefore, a ceRNA network was constructed to view the potential roles of the m6A-related lncRNAs in AML. A total of 9 lncRNAs, 10 microRNAs and 271 mRNAs were used to construct the network (Figure 10A). Two significant modules, dominated by FOS and NOTCH1 nodes, were identified from the PPI network, and 10 hub genes were extracted (Figure 10B). Moreover, the functional enrichment analysis with 271 target mRNAs showed that the target genes were enriched in regulation of protein serine/threonine kinase activity, protein autophosphorylation, positive regulation of catabolic process, response to transforming growth factor β and epithelial cell proliferation and migration (GO Biological Processes, Figure 10C). MAPK, PI3K-Akt, Rap1, Ras, TGF-β, Estrogen, FoxO signaling pathway, and microRNA in cancer (KEGG Pathway, Figure 10D). The results showed other m6A-related lncRNAs also play critical roles during tumor progression, and provide new insights to study the potential roles and mechanism of m6A-related lncRNAs in AML.

## Discussion

To the best of our knowledge, AML is a deadly disease associated with poor outcomes despite advancements in targeted molecular and immunotherapy (Short et al., 2018). The m6A is the most abundant epigenetic modification of mRNA and lncRNAs and plays important roles in many biological processes (Wei et al., 2017). The lncRNAs do not possess protein coding ability but participate in a plethora of cellular functions (Ponting et al., 2009; Morlando and Fatica, 2018). The m6A modifications of lncRNAs are associated with the development, occurrence, and prognosis of a variety of tumors and other diseases, including AML (Coker et al., 2019), lncRNAs can also affect tumor invasive progression by targeting m6A regulators as competitive endogenous RNAs (ceRNAs) (Tu et al., 2020). Both m6A and lncRNAs are important regulators of AML (Zimta et al., 2019; Zheng and Gong, 2021). However, the potential roles of m6A-related lncRNAs in AML remains unclear. Thus, based on the data from the TCGA dataset, we developed a novel m6A-related lncRNAs signature to accurately predict the prognoses in AML.

A total of 144 AML samples were included to explore the prognostic value of m6A-related lncRNAs. Seven of 15 m6A-related lncRNAs with prognostic value were used to establish a signature to predict the OS of AML patients. The high-risk group was significantly associated with a poor prognosis. Furthermore, multivariate Cox regression analysis illustrated that the signature acts as an independent prognostic factor in AML, which was consistent with the results of ROC curve analysis.

Among the lncRNAs in the prognostic signature, USP30-AS1 has been reported to be related to autophagy and immunity in bladder cancer (Wan et al., 2021), cervical cancer (Chen et al., 2020) and melanoma (Ding et al., 2021). MIR133A1HG was also reported to be one of the lncRNAs in the autophagy-related signature for AML (Zhao et al., 2021). However, the underlying molecular biological mechanism has not been elucidated. Notably, AC114271.2, AF064858.8, RP11-22L13.1, RP11-544A12.4 and MIR181A1HG were revealed for the first time. The correlation analysis revealed that two of seven lncRNAs, MIR133A1HG and RP11-544A12.4, have significant correlation with the expression of almost all m6A regulators, and that MIR181A1HG, AC114271.2 and USP30-AS1 were related to some m6A regulators, including m6A writers, erasers, and readers. Furthermore, AF064858.8 is related to some m6A RNA writers and readers, while RP11-22L13.1 is related to m6A RNA readers IGF2BP2 and IGF2BP3. However, the specific mechanism on how lncRNA regulates m6A remains to be further studied (Supplementary Figure S3). The results of the pan-cancer analysis showed that USP30-AS1, AF064858.8, MIR133A1HG, RP11-544A12.4 and MIR181A1HG were specifically expressed in AML, which indicated that these lncRNAs play a crucial role in its carcinogenesis (Supplementary Figures S4, S5).

GO and KEGG revealed that cancer hallmark-related and malignancy-related pathways were more enriched in the high-risk group. In addition, we found that T cell activation was also enriched, indicating that m6A-related lncRNAs may affect the prognosis of patients through immune mechanisms, which is consistent with the results of the previous studies (Xu et al., 2021; Zhang et al., 2021). The infiltrating immune cells in the tumor microenvironment (TME), particularly T cells, are key mediators of tumor destruction and play important roles in immunotherapy (Lei et al., 2020). m6A has been reported to regulate the maturation and neoantigen presentation involved in the immunotherapeutic response (Li et al., 2021). Nevertheless, the potential function and prognostic value of m6A-related lncRNAs in mediating immunotherapeutic response and prognosis in AML remain to be characterized. The TIDE prediction score is a successfully validated computational framework for immunotherapy prediction (Jiang et al., 2018). Correspondingly, the TIDE algorithm was also performed to predict the correlation between the risk score and the immunotherapeutic response, the results indicated that risk scores of the no-response group were higher than those of the response group. Obviously, this signature was significantly correlated with immunity and a potential biomarker for predicting the response to immunotherapy in AML.

Seven of the 15 m6A-related lncRNAs with prognostic value were used to construct the prognostic signature. This does not necessarily mean that these 7 lncRNAs are more important than other m6A-related lncRNAs; rather, it indicates that the combination of these 7 m6A-related lncRNAs can adequately predict AML prognosis. The results of the ceRNA network and functional enrichment analysis showed that other m6A-related lncRNAs (SENCR, PSMB8-AS1, ITGB2-AS1, RP11-89N17.1, RP11-483I13.5 and LINC01547) also play critical roles during tumor progression by regulating the expression of important genes such as PTEN, VEGFA, MAPK1, IGF1, etc. These genes may provide us with new therapeutic targets for AML patients.

In conclusion, we first constructed a 7 m6A-related lncRNA risk signature to predict the prognosis of AML and verified the predictive reliability and sensitivity of the signature. We also explored the distinct molecular landscape classified by the signature, including biological processes, pathways, correlation with immune therapies, potential targets, and provided new insights into the potential roles and mechanisms of m6A-related lncRNAs in AML.

However, our study has several limitations. First, we used the data from the public dataset TCGA to construct and validate the signature. There was no suitable external database on AML to assess the reliability of the signature. Additionally, functional analyses and mechanistic studies of the signature were not carried out.

## Data Availability

The datasets presented in this study can be found in online repositories. The names of the repository/repositories and accession number(s) can be found in the article/Supplementary Material.

## Supplementary Material

Supplementary Figure S1 Kaplan–Meier survival curves for the 7 signature lncRNAs in AML.

Supplementary Figure S2 The most pronounced differential biological processes between the high- and low-risk groups explored by GSEA.

Supplementary Figure S3 The correlation analysis between seven lncRNAs and 21 m6A genes.

Supplementary Figure S4 The expressions of 7 signature lncRNAs in pan-cancer from TCGA.

Supplementary Figure S5 The expressions of 7 signature lncRNAs in AML and normal tissues analyzed by GEPIA.

Supplementary Table S1 The clinical characteristics for AML cases.

Supplementary Table S2 Primer sequences for qRT-PCR.

## References

[B1] Cao G. Li H.-B. Yin Z. Flavell R. A. (2016). Recent Advances in Dynamic M 6 A RNA Modification. Open Biol. 6 (4), 160003. 10.1098/rsob.160003 27249342 PMC4852458

[B2] Chandra Gupta S. Nandan Tripathi Y. (2017). Potential of Long Non-coding RNAs in Cancer Patients: From Biomarkers to Therapeutic Targets. Int. J. Cancer 140 (9), 1955–1967. 10.1002/ijc.30546 27925173

[B3] Chen L. Hu N. Wang C. Zhao H. (2020a). HOTAIRM1 Knockdown Enhances Cytarabine-Induced Cytotoxicity by Suppression of Glycolysis through the Wnt/β-Catenin/PFKP Pathway in Acute Myeloid Leukemia Cells. Arch. Biochem. Biophys. 680, 108244. 10.1016/j.abb.2019.108244 31904363

[B4] Chen P. Gao Y. Ouyang S. Wei L. Zhou M. You H. (2020b). A Prognostic Model Based on Immune-Related Long Non-coding RNAs for Patients with Cervical Cancer. Front. Pharmacol. 11, 585255. 10.3389/fphar.2020.585255 33328990 PMC7734341

[B5] Chen Y. Lin Y. Shu Y. He J. Gao W. (2020c). Interaction between N6-Methyladenosine (m6A) Modification and Noncoding RNAs in Cancer. Mol. Cancer 19 (1), 94. 10.1186/s12943-020-01207-4 32443966 PMC7243333

[B6] Choi W. Heo M. Y. Kim S. Y. Wee J.-H. Kim Y.-H. Min J. (2020). Encapsulation of Daunorubicin into Saccharomyces Cerevisiae-Derived Lysosome as Drug Delivery Vehicles for Acute Myeloid Leukemia (AML) Treatment. J. Biotechnol. 308, 118–123. 10.1016/j.jbiotec.2019.12.008 31846628

[B7] Coker H. Wei G. Brockdorff N. (2019). m6A Modification of Non-coding RNA and the Control of Mammalian Gene Expression. Biochim. Biophys. Acta (Bba) - Gene Regul. Mech. 1862 (3), 310–318. 10.1016/j.bbagrm.2018.12.002 30550772

[B8] Cui C. Wang Y. Gong W. He H. Zhang H. Shi W. (2021). Long Non-coding RNA LINC00152 Regulates Self-Renewal of Leukemia Stem Cells and Induces Chemo-Resistance in Acute Myeloid Leukemia. Front. Oncol. 11, 694021. 10.3389/fonc.2021.694021 34295821 PMC8290167

[B9] Dai D. Wang H. Zhu L. Jin H. Wang X. (2018). N6-methyladenosine Links RNA Metabolism to Cancer Progression. Cell Death Dis. 9 (2), 124. 10.1038/s41419-017-0129-x 29374143 PMC5833385

[B10] Deng X. Su R. Weng H. Huang H. Li Z. Chen J. (2018). RNA N6-Methyladenosine Modification in Cancers: Current Status and Perspectives. Cell Res. 28 (5), 507–517. 10.1038/s41422-018-0034-6 29686311 PMC5951805

[B11] Ding Y. Li T. Li M. Tayier T. Zhang M. Chen L. (2021). A Novel Autophagy-Related lncRNA Gene Signature to Improve the Prognosis of Patients with Melanoma. Biomed. Res. Int. 2021, 1–12. 10.1155/2021/8848227 PMC8238568 34250091

[B12] Fernando T. R. Contreras J. R. Zampini M. Rodriguez-Malave N. I. Alberti M. O. Anguiano J. (2017). The lncRNA CASC15 Regulates SOX4 Expression in RUNX1-Rearranged Acute Leukemia. Mol. Cancer 16 (1), 126. 10.1186/s12943-017-0692-x 28724437 PMC5517805

[B13] Gibb E. A. Brown C. J. Lam W. L. (2011). The Functional Role of Long Non-coding RNA in Human Carcinomas. Mol. Cancer 10, 38. 10.1186/1476-4598-10-38 21489289 PMC3098824

[B14] Groff A. F. Sanchez-Gomez D. B. Soruco M. M. L. Gerhardinger C. Barutcu A. R. Li E. (2016). *In Vivo* Characterization of Linc-P21 Reveals Functional Cis -Regulatory DNA Elements. Cel Rep. 16 (8), 2178–2186. 10.1016/j.celrep.2016.07.050 PMC5014909 27524623

[B15] Huang Y. Su R. Sheng Y. Dong L. Dong Z. Xu H. (2019). Small-Molecule Targeting of Oncogenic FTO Demethylase in Acute Myeloid Leukemia. Cancer Cell 35 (4), 677–691. e610. 10.1016/j.ccell.2019.03.006 30991027 PMC6812656

[B16] Jiang P. Gu S. Pan D. Fu J. Sahu A. Hu X. (2018). Signatures of T Cell Dysfunction and Exclusion Predict Cancer Immunotherapy Response. Nat. Med. 24 (10), 1550–1558. 10.1038/s41591-018-0136-1 30127393 PMC6487502

[B17] Koenig K. Mims A. (2020). Relapsed or Primary Refractory AML. Curr. Opin. Hematol. 27 (2), 108–114. 10.1097/MOH.0000000000000561 31904664 PMC7015186

[B18] Lei X. Lei Y. Li J.-K. Du W.-X. Li R.-G. Yang J. (2020). Immune Cells within the Tumor Microenvironment: Biological Functions and Roles in Cancer Immunotherapy. Cancer Lett. 470, 126–133. 10.1016/j.canlet.2019.11.009 31730903

[B19] Li J. Wang M. Chen X. (2020). Long Non-coding RNA UCA1 Modulates Cell Proliferation and Apoptosis by Regulating miR-296-3p/Myc axis in Acute Myeloid Leukemia. Cell Cycle 19 (12), 1454–1465. 10.1080/15384101.2020.1750814 32286143 PMC7469675

[B20] Li M. Zha X. Wang S. (2021). The Role of N6-Methyladenosine mRNA in the Tumor Microenvironment. Biochim. Biophys. Acta (Bba) - Rev. Cancer 1875 (2), 188522. 10.1016/j.bbcan.2021.188522 33545295

[B21] Long Y. Wang X. Youmans D. T. Cech T. R. (2017). How Do lncRNAs Regulate Transcription? Sci. Adv. 3 (9), eaao2110. 10.1126/sciadv.aao2110 28959731 PMC5617379

[B22] Morlando M. Fatica A. (2018). Alteration of Epigenetic Regulation by Long Noncoding RNAs in Cancer. Ijms 19 (2), 570. 10.3390/ijms19020570 PMC5855792 29443889

[B23] Paris J. Morgan M. Campos J. Spencer G. J. Shmakova A. Ivanova I. (2019). Targeting the RNA m6A Reader YTHDF2 Selectively Compromises Cancer Stem Cells in Acute Myeloid Leukemia. Cell Stem Cell 25 (1), 137–148. e136. 10.1016/j.stem.2019.03.021 31031138 PMC6617387

[B24] Ponting C. P. Oliver P. L. Reik W. (2009). Evolution and Functions of Long Noncoding RNAs. Cell 136 (4), 629–641. 10.1016/j.cell.2009.02.006 19239885

[B25] Shen C. Sheng Y. Zhu A. C. Robinson S. Jiang X. Dong L. (2020). RNA Demethylase ALKBH5 Selectively Promotes Tumorigenesis and Cancer Stem Cell Self-Renewal in Acute Myeloid Leukemia. Cell Stem Cell 27 (1), 64–80. e69. 10.1016/j.stem.2020.04.009 32402250 PMC7335338

[B26] Short N. J. Rytting M. E. Cortes J. E. (2018). Acute Myeloid Leukaemia. The Lancet 392 (10147), 593–606. 10.1016/S0140-6736(18)31041-9 PMC10230947 30078459

[B27] Tay Y. Rinn J. Pandolfi P. P. (2014). The Multilayered Complexity of ceRNA Crosstalk and Competition. Nature 505 (7483), 344–352. 10.1038/nature12986 24429633 PMC4113481

[B28] Tu Z. Wu L. Wang P. Hu Q. Tao C. Li K. (2020). N6-Methylandenosine-Related lncRNAs Are Potential Biomarkers for Predicting the Overall Survival of Lower-Grade Glioma Patients. Front. Cel Dev. Biol. 8, 642. 10.3389/fcell.2020.00642 PMC7390977 32793593

[B29] Vu L. P. Pickering B. F. Cheng Y. Zaccara S. Nguyen D. Minuesa G. (2017). The N6-Methyladenosine (m6A)-Forming Enzyme METTL3 Controls Myeloid Differentiation of normal Hematopoietic and Leukemia Cells. Nat. Med. 23 (11), 1369–1376. 10.1038/nm.4416 28920958 PMC5677536

[B30] Wan J. Guo C. Fang H. Xu Z. Hu Y. Luo Y. (2021). Autophagy-Related Long Non-coding RNA Is a Prognostic Indicator for Bladder Cancer. Front. Oncol. 11, 647236. 10.3389/fonc.2021.647236 33869042 PMC8049181

[B31] Wei W. Ji X. Guo X. Ji S. (2017). Regulatory Role of N6-Methyladenosine (m6A) Methylation in RNA Processing and Human Diseases. J. Cel. Biochem. 118 (9), 2534–2543. 10.1002/jcb.25967 28256005

[B32] Weng H. Huang H. Wu H. Qin X. Zhao B. S. Dong L. (2018). METTL14 Inhibits Hematopoietic Stem/Progenitor Differentiation and Promotes Leukemogenesis via mRNA m6A Modification. Cell Stem Cell 22 (2), 191–205. e199. 10.1016/j.stem.2017.11.016 29290617 PMC5860916

[B33] Xu F. Huang X. Li Y. Chen Y. Lin L. (2021). m6A-related lncRNAs Are Potential Biomarkers for Predicting Prognoses and Immune Responses in Patients with LUAD. Mol. Ther. - Nucleic Acids 24, 780–791. 10.1016/j.omtn.2021.04.003 33996259 PMC8094594

[B34] Yu G. Wang L.-G. Han Y. He Q.-Y. (2012). clusterProfiler: an R Package for Comparing Biological Themes Among Gene Clusters. OMICS: A J. Integr. Biol. 16 (5), 284–287. 10.1089/omi.2011.0118 PMC3339379 22455463

[B35] Zhang P. Liu G. Lu L. (2021). N6-Methylandenosine-Related lncRNA Signature Is a Novel Biomarkers of Prognosis and Immune Response in Colon Adenocarcinoma Patients. Front. Cel Dev. Biol. 9, 703629. 10.3389/fcell.2021.703629 PMC8321625 34336856

[B36] Zhang T.-j. Zhou J.-d. Zhang W. Lin J. Ma J.-c. Wen X.-m. (2018). H19 Overexpression Promotes Leukemogenesis and Predicts Unfavorable Prognosis in Acute Myeloid Leukemia. Clin. Epigenet 10, 47. 10.1186/s13148-018-0486-z PMC5891930 29643943

[B37] Zhao C. Wang Y. Tu F. Zhao S. Ye X. Liu J. (2021). A Prognostic Autophagy-Related Long Non-coding RNA (ARlncRNA) Signature in Acute Myeloid Leukemia (AML). Front. Genet. 12, 681867. 10.3389/fgene.2021.681867 34276784 PMC8278057

[B38] Zheng X. Gong Y. (2021). Functions of RNA N6-Methyladenosine Modification in Acute Myeloid Leukemia. Biomark Res. 9 (1), 36. 10.1186/s40364-021-00293-w 34001273 PMC8130309

[B39] Zimta A.-A. Tomuleasa C. Sahnoune I. Calin G. A. Berindan-Neagoe I. (2019). Long Non-coding RNAs in Myeloid Malignancies. Front. Oncol. 9, 1048. 10.3389/fonc.2019.01048 31681586 PMC6813191

