# Supplementary material for: Identification of m6A-Related lncRNAs Associated With Prognoses and Immune Responses in Acute Myeloid Leukemia
Source: Front Cell Dev Biol. 2021 Nov 16;9:770451. doi: 10.3389/fcell.2021.770451 (PMC8637120; doi:10.3389/fcell.2021.770451)
Supplement: Supplementary file 1 [file DataSheet7.pdf]

Table S2 Primer sequences for qRT-PCR

Gene name		Sequences
GAPDH	Forward	5’-GCACCGTCAAGGCTGAGAAC-3’
	Reverse	5’-TGGTGAAGACGCCAGTGGA-3’
USP30-AS1	Forward	5’-GAGCAATAGCTGACGGACCA-3’
	Reverse	5’-TGAAAACCAAGCAGCCCCAG-3’
AC114271.2	Forward	5’-GATGTGAACAGCGTTGCGTT-3’
	Reverse	5’-CAGTCCCCAGACTTCTGCAAT-3’
AF064858.8	Forward	5’-GTAGACGGACCTCAGCACAG-3’
	Reverse	5’-AGGGATGACACGCAGCTAAG-3’
RP11-22L13.1	Forward	5’-AAGGTGTCTACCGTGGGACT-3’
	Reverse	5’-CCTTGATCTGAGCCTCGCTT-3’
MIR181A1HG	Forward	5’-TCTCCGGCATGAAAACAGGG-3’
	Reverse	5’-TTAGGGTACCACCAACCAAGC-3’
RP11-544A12.4	Forward	5’-GACGTTTCCAGGGTCCCATC-3’
	Reverse	5’-AGCTGCTTTTTCTTGTATCTGGT-3’
MIR133A1HG	Forward	5’-ATGCTGATTCTGGTGCTTGC-3’
	Reverse	5’-TCAGCAGATGCCCAGAGTTT-3’
