# Supplementary figures and images for: Identification of m6A-Related lncRNAs Associated With Prognoses and Immune Responses in Acute Myeloid Leukemia
Source: Front Cell Dev Biol. 2021 Nov 16;9:770451. doi: 10.3389/fcell.2021.770451 (PMC8637120; doi:10.3389/fcell.2021.770451)

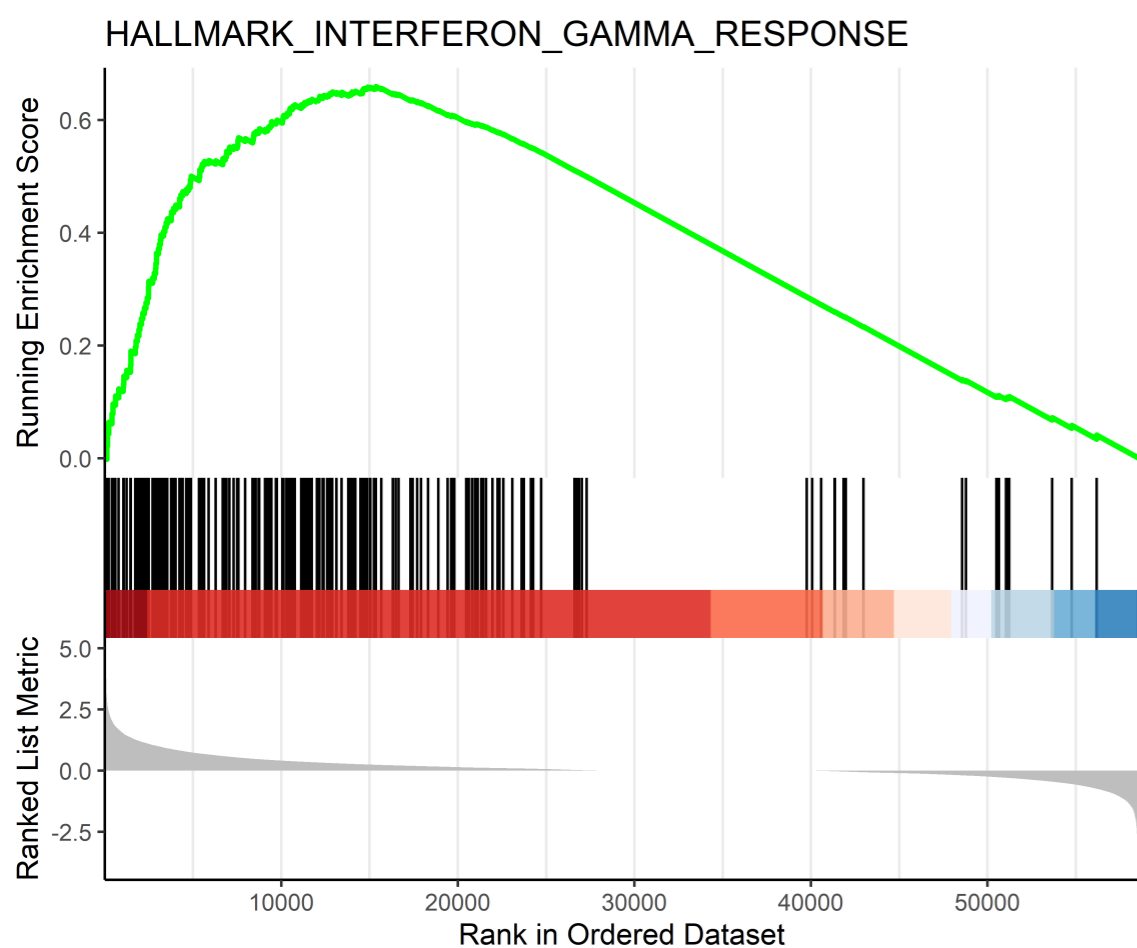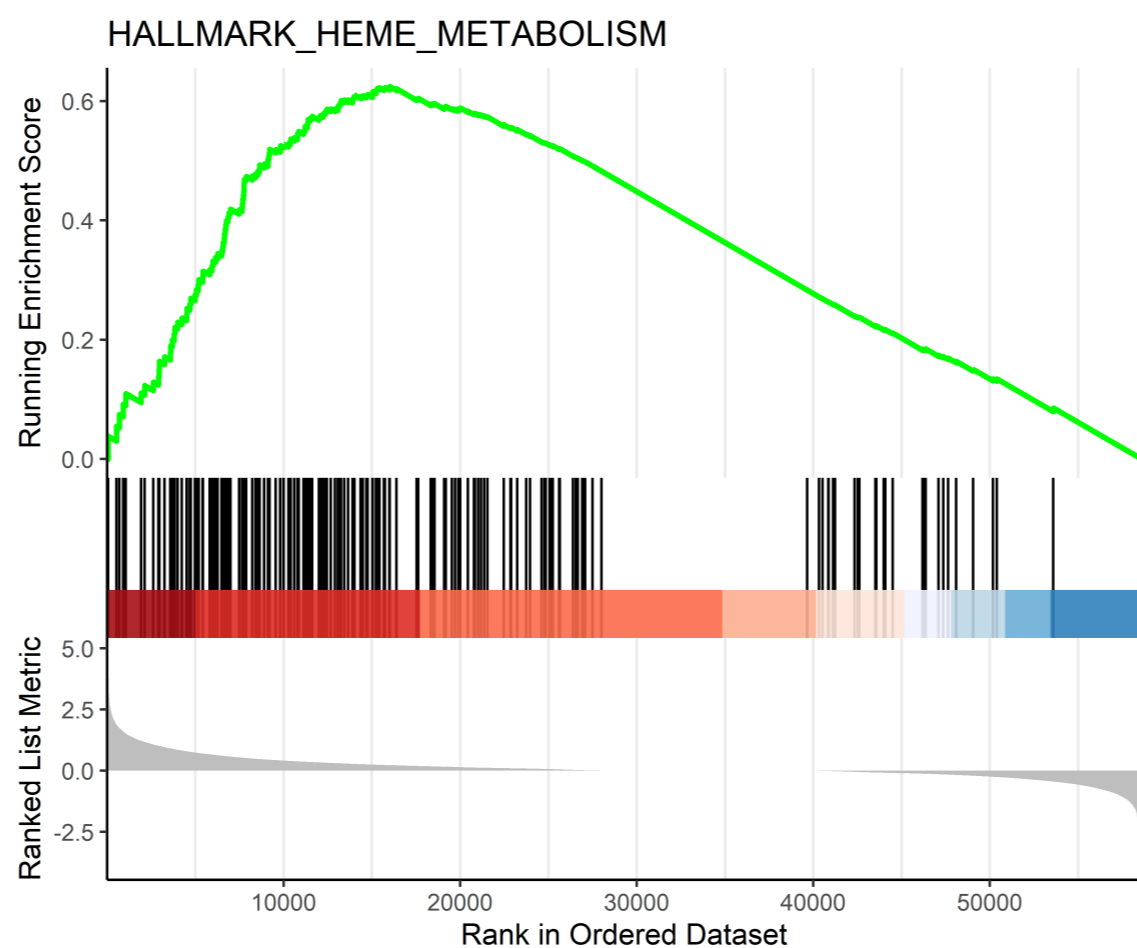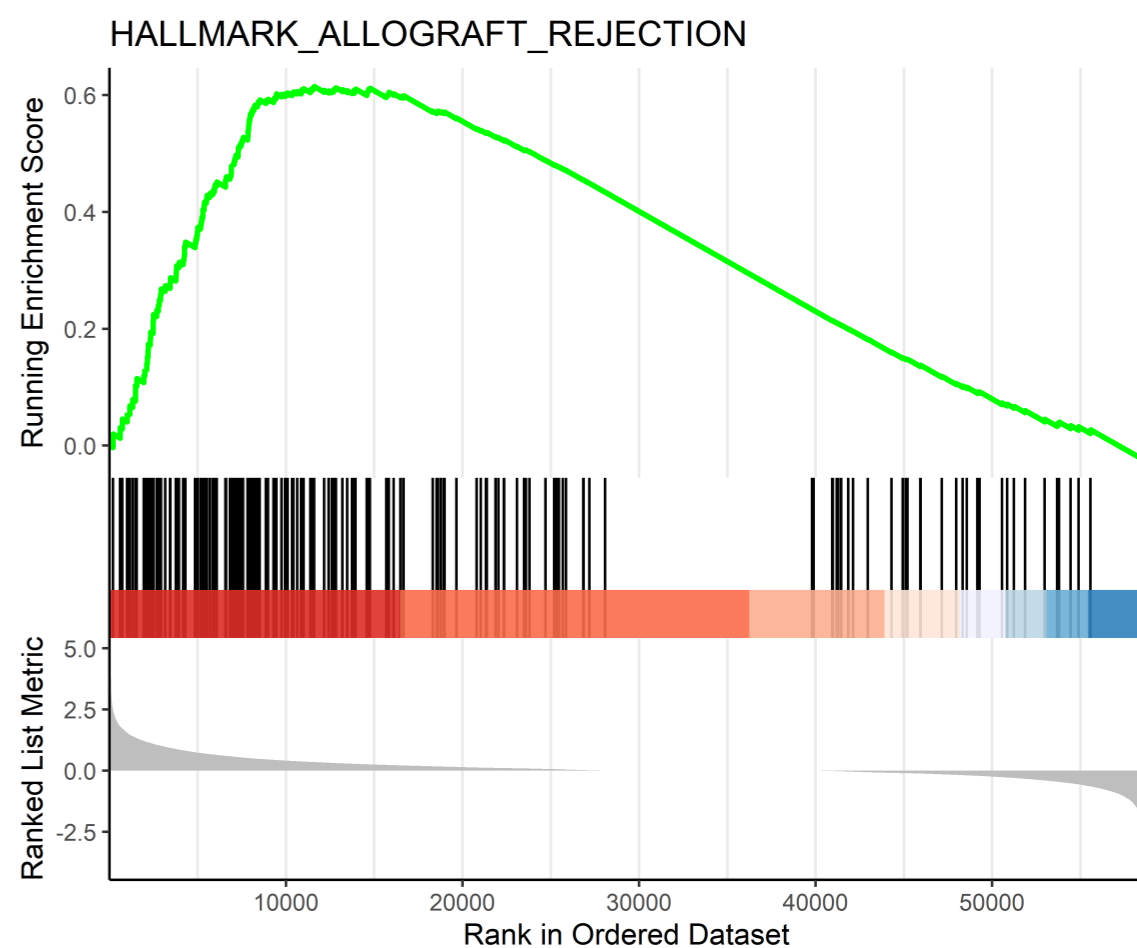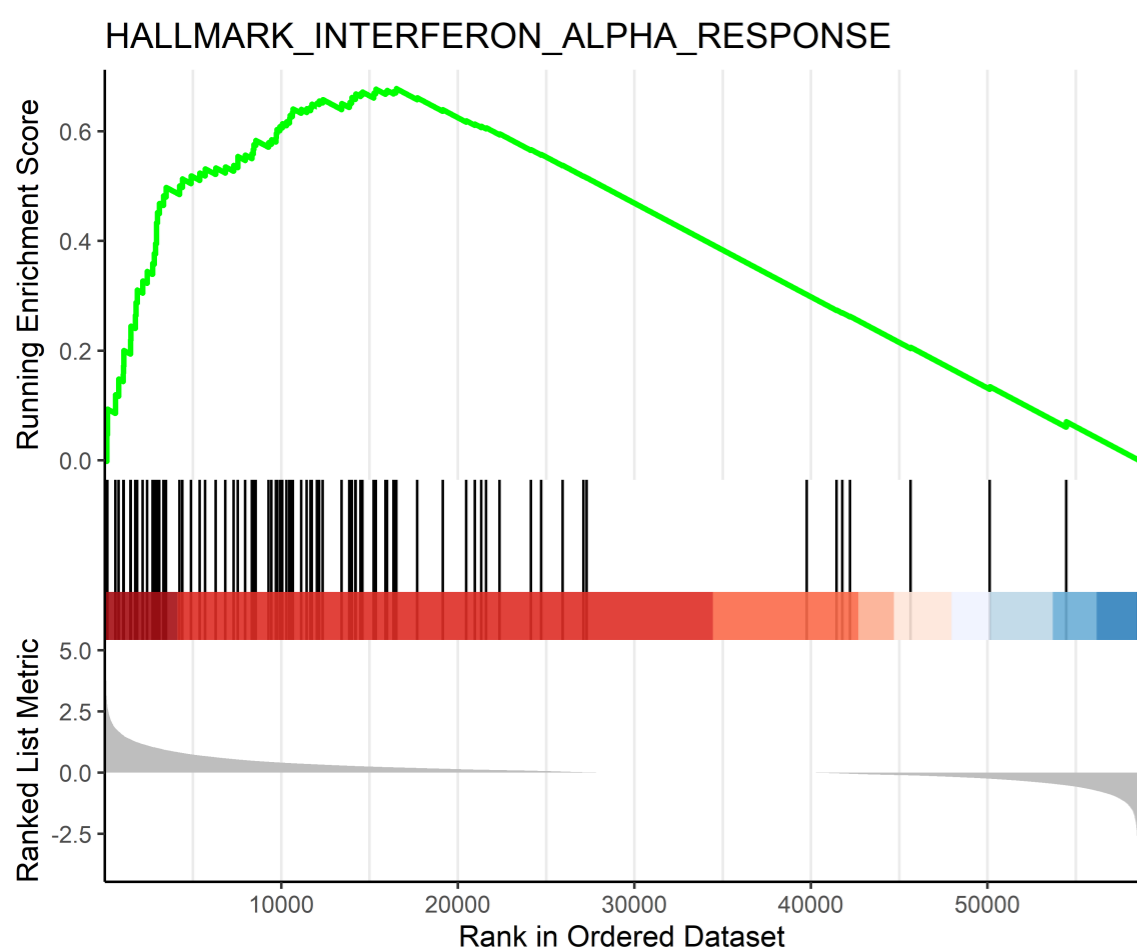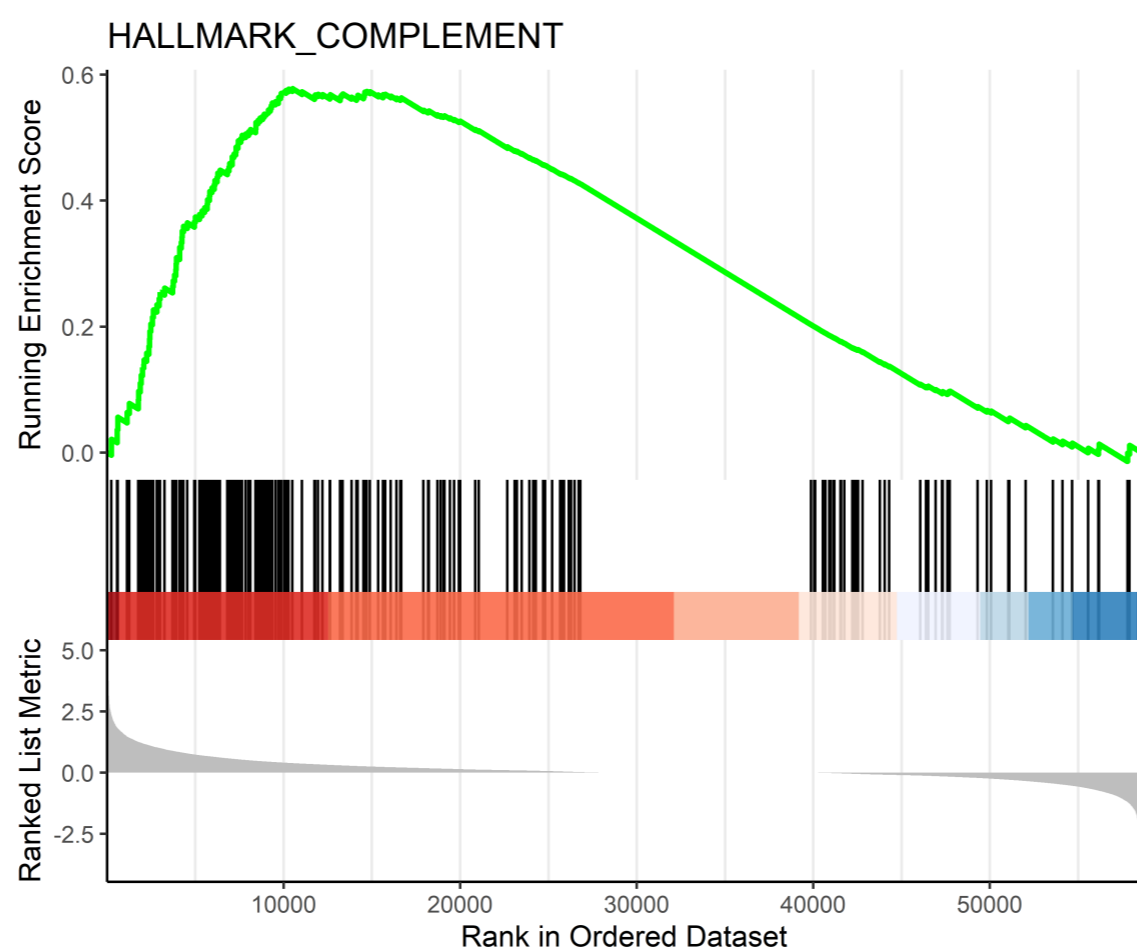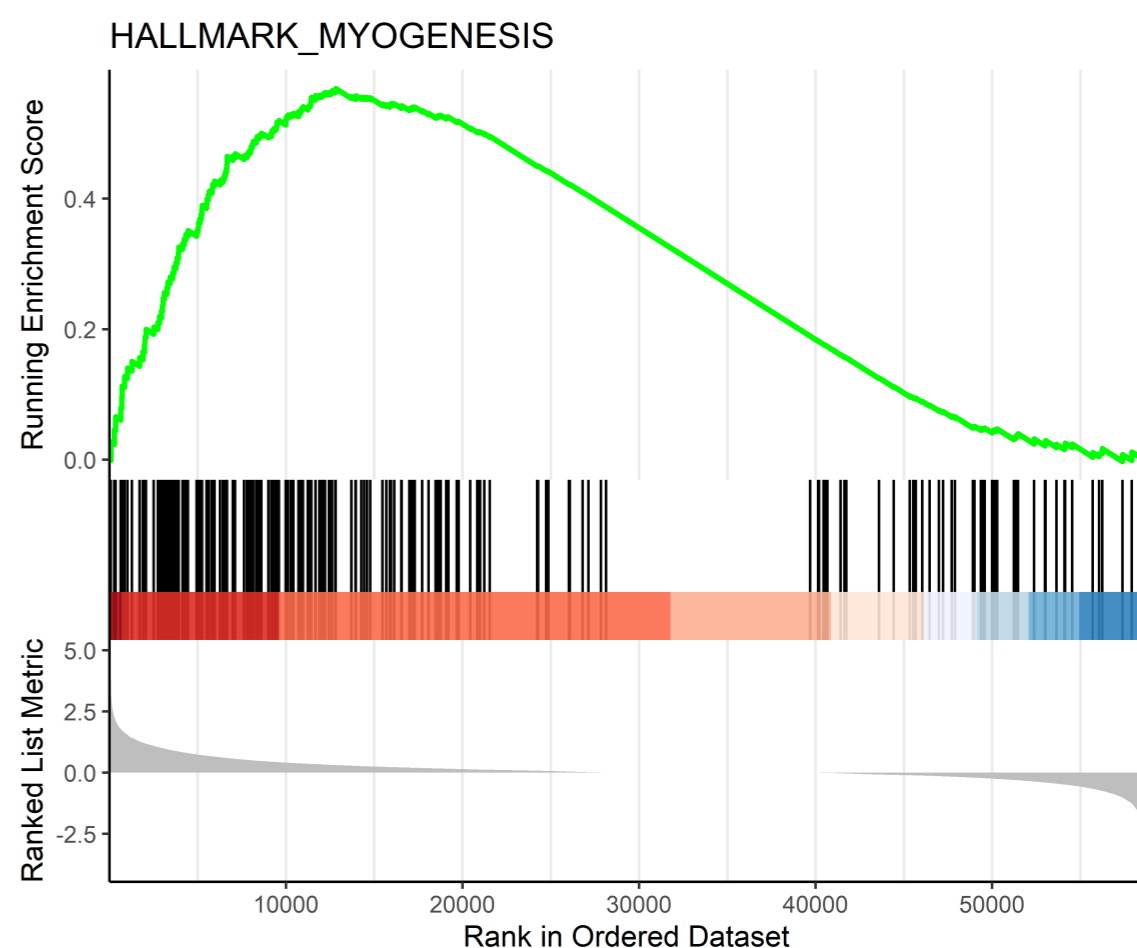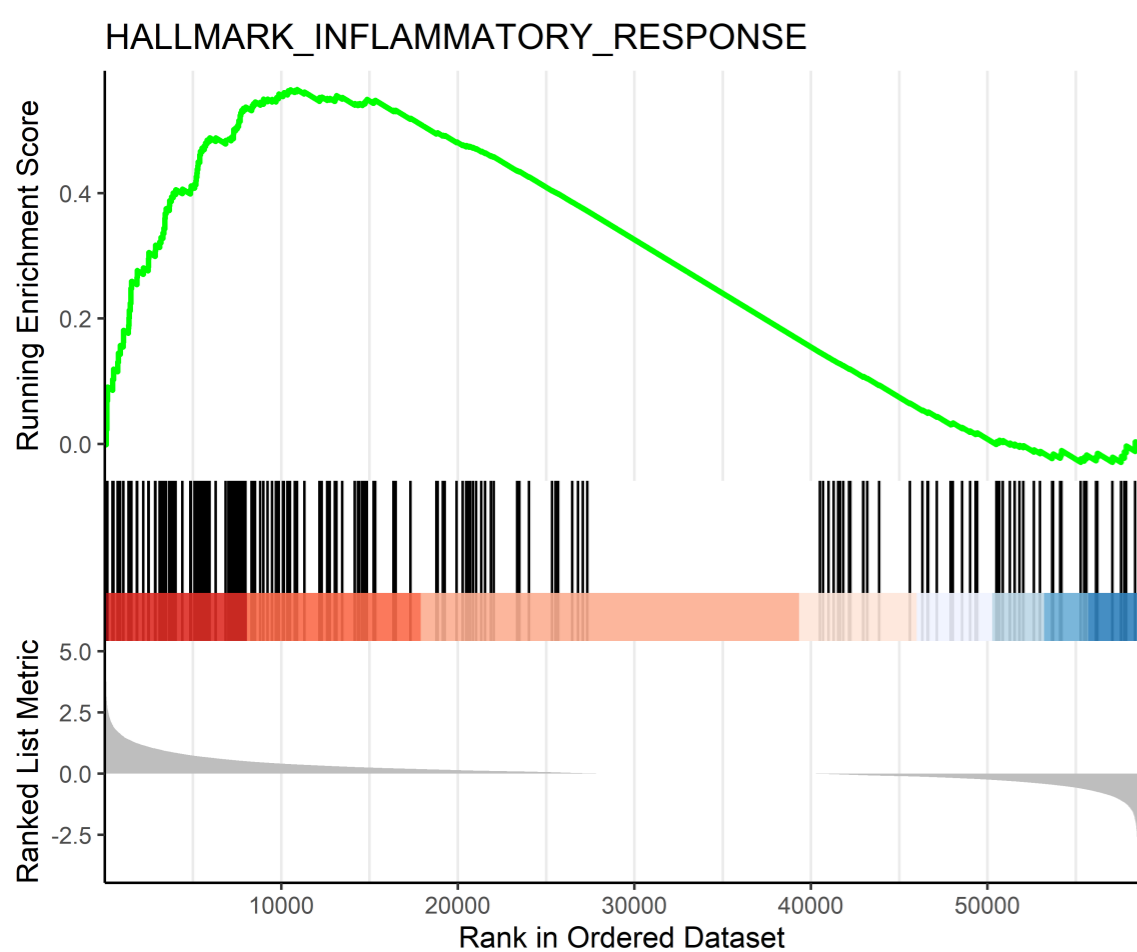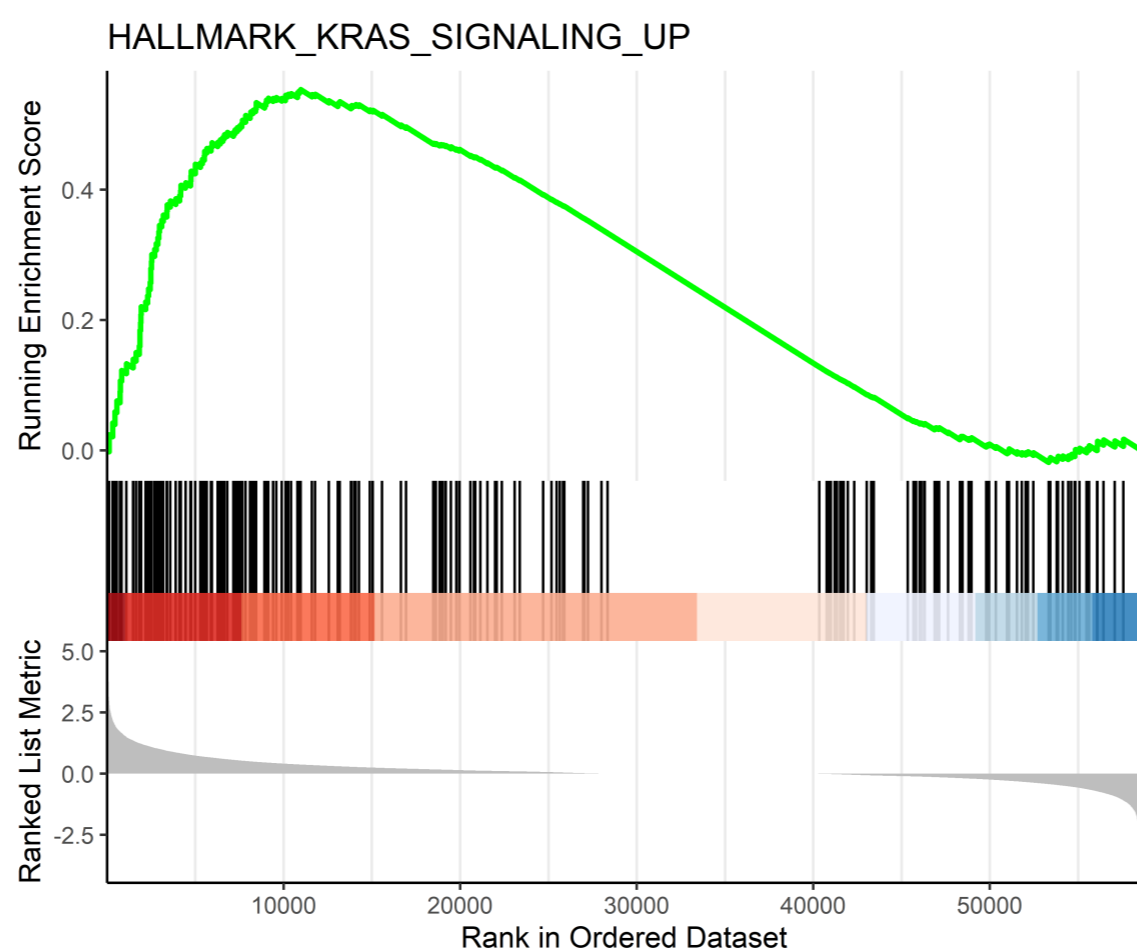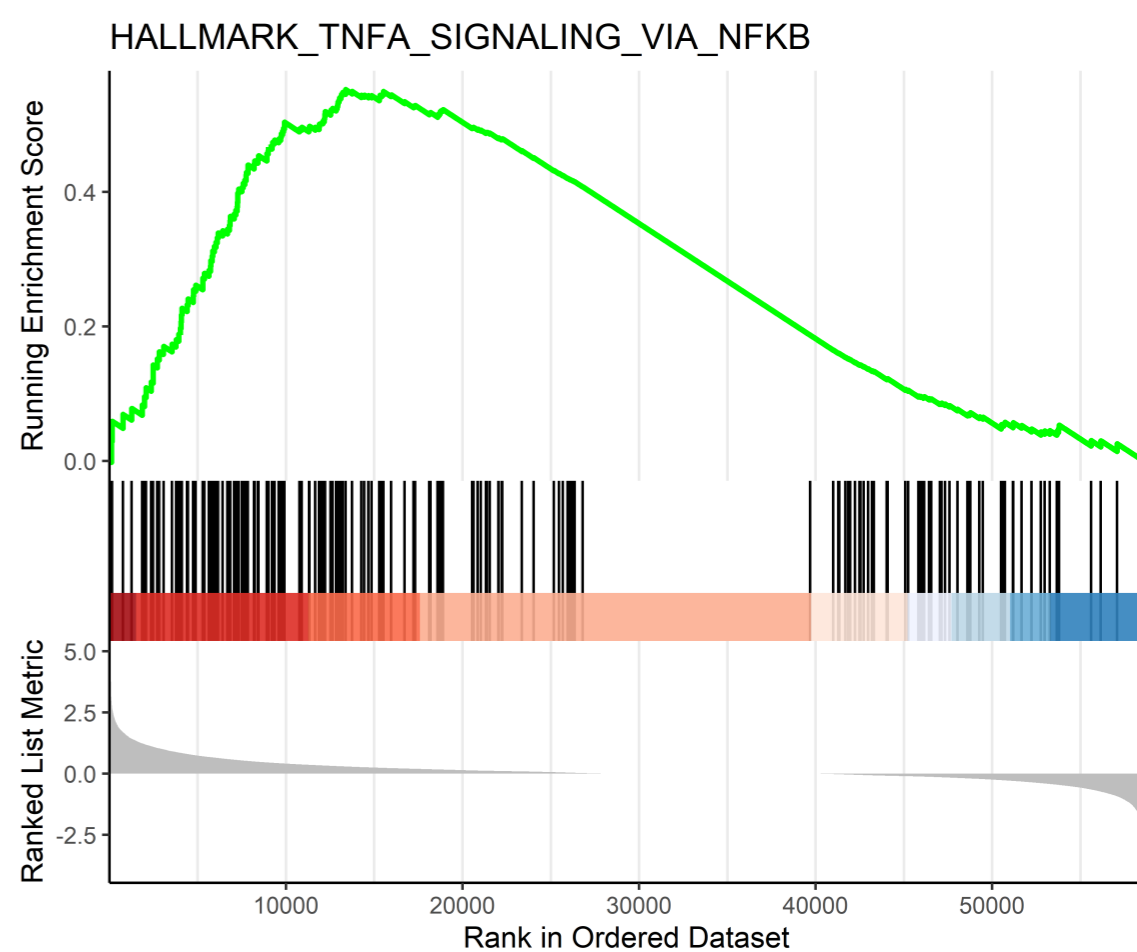

Supplement: Supplementary file 2 [file DataSheet2.pdf]

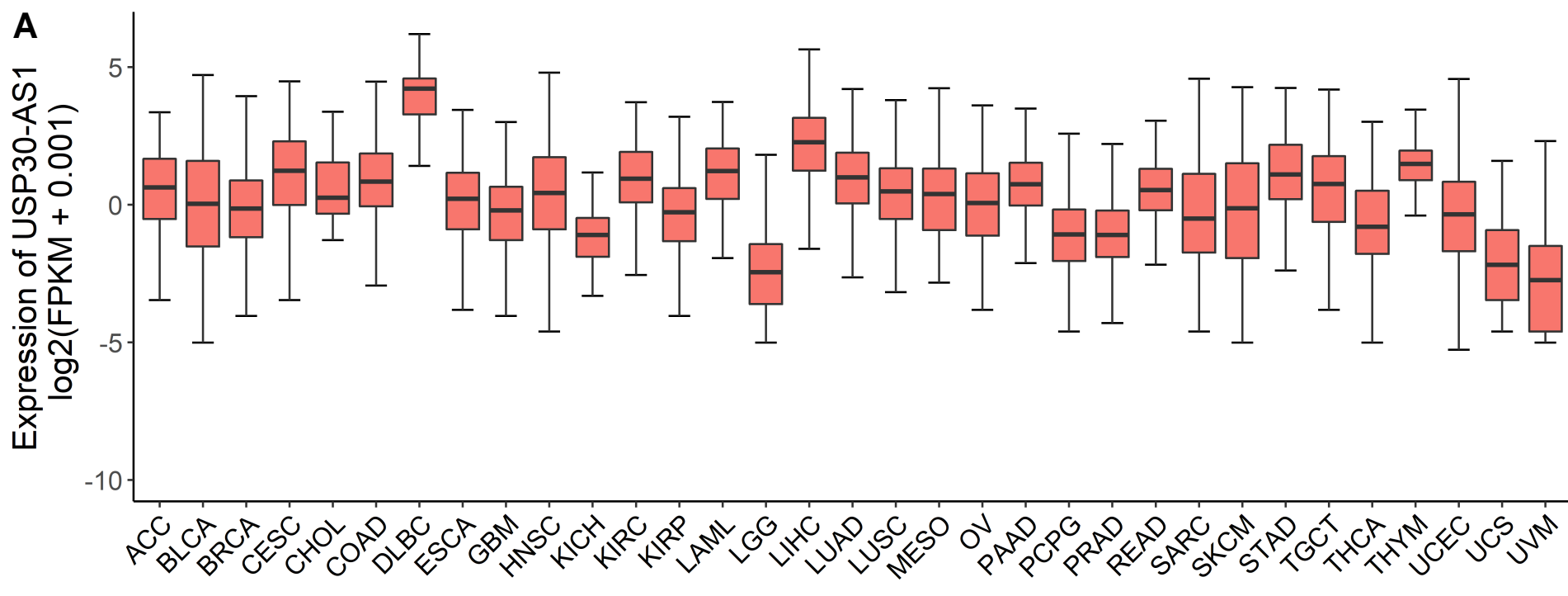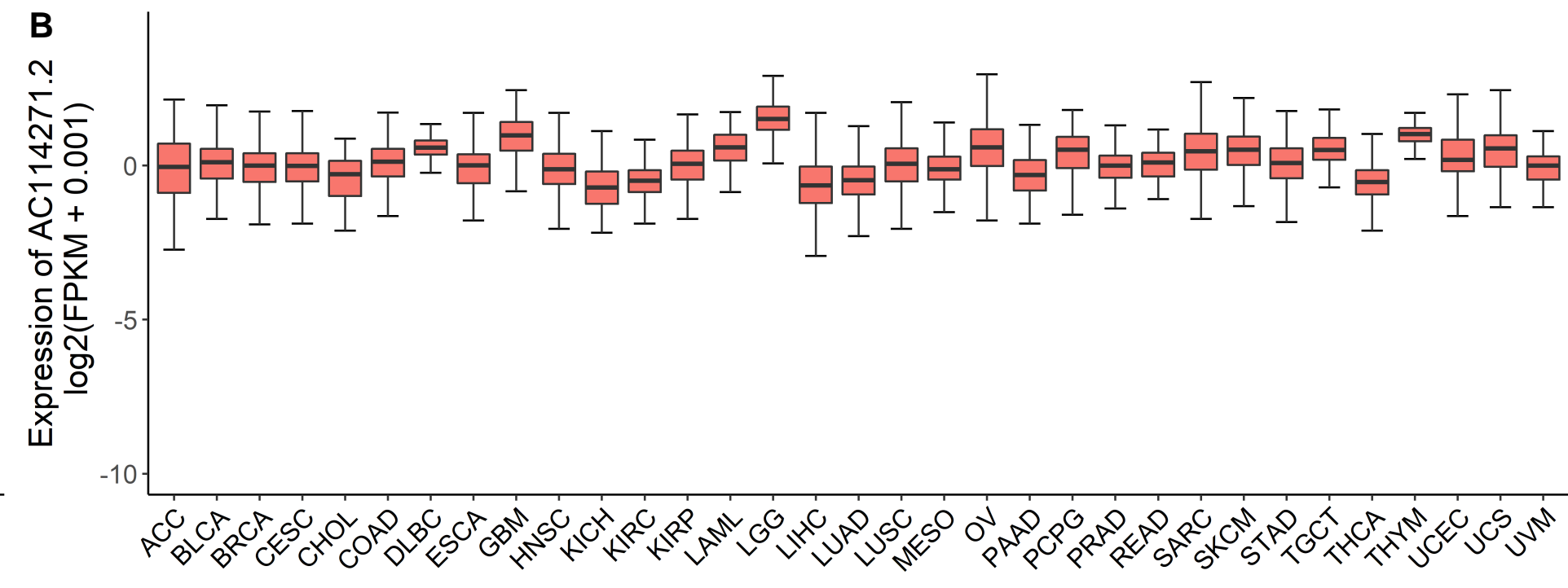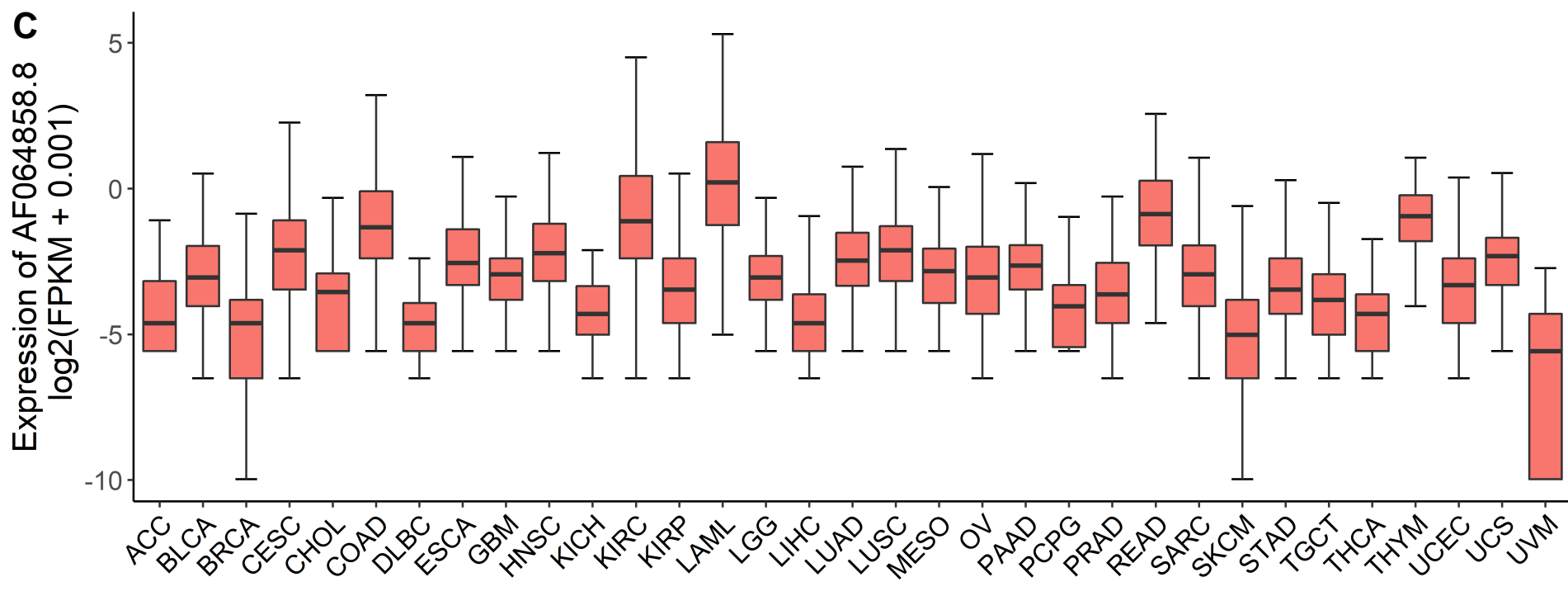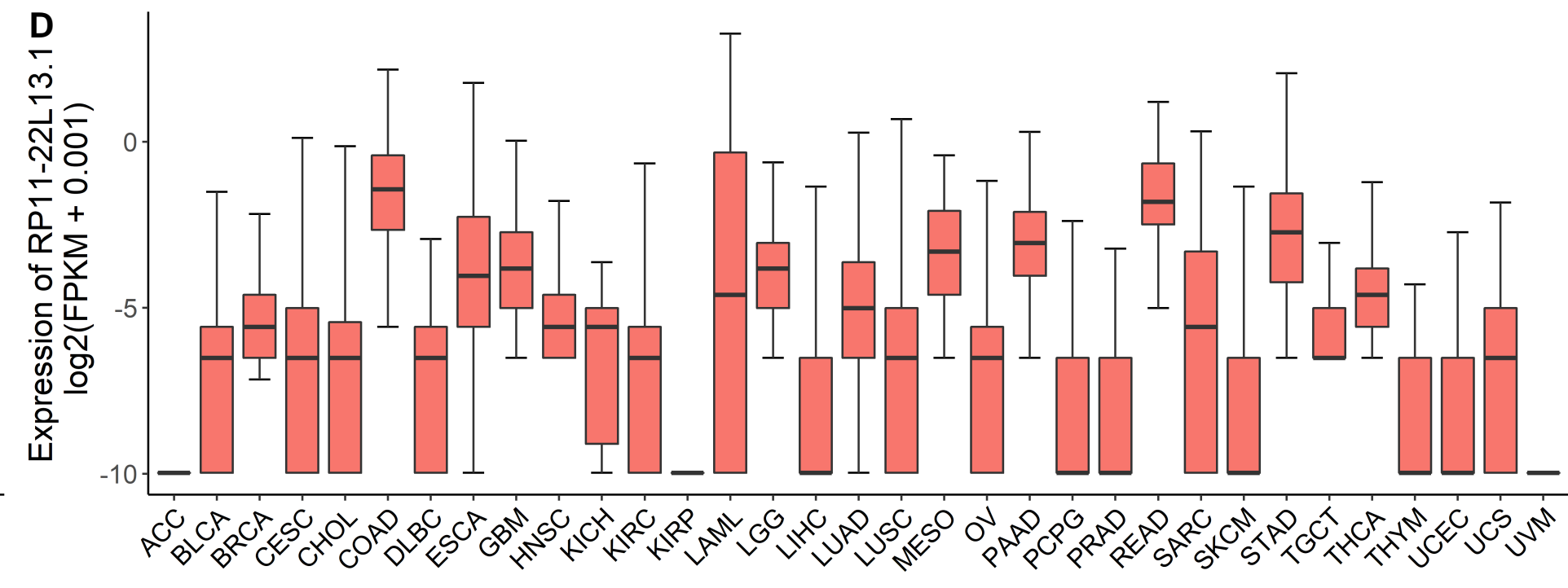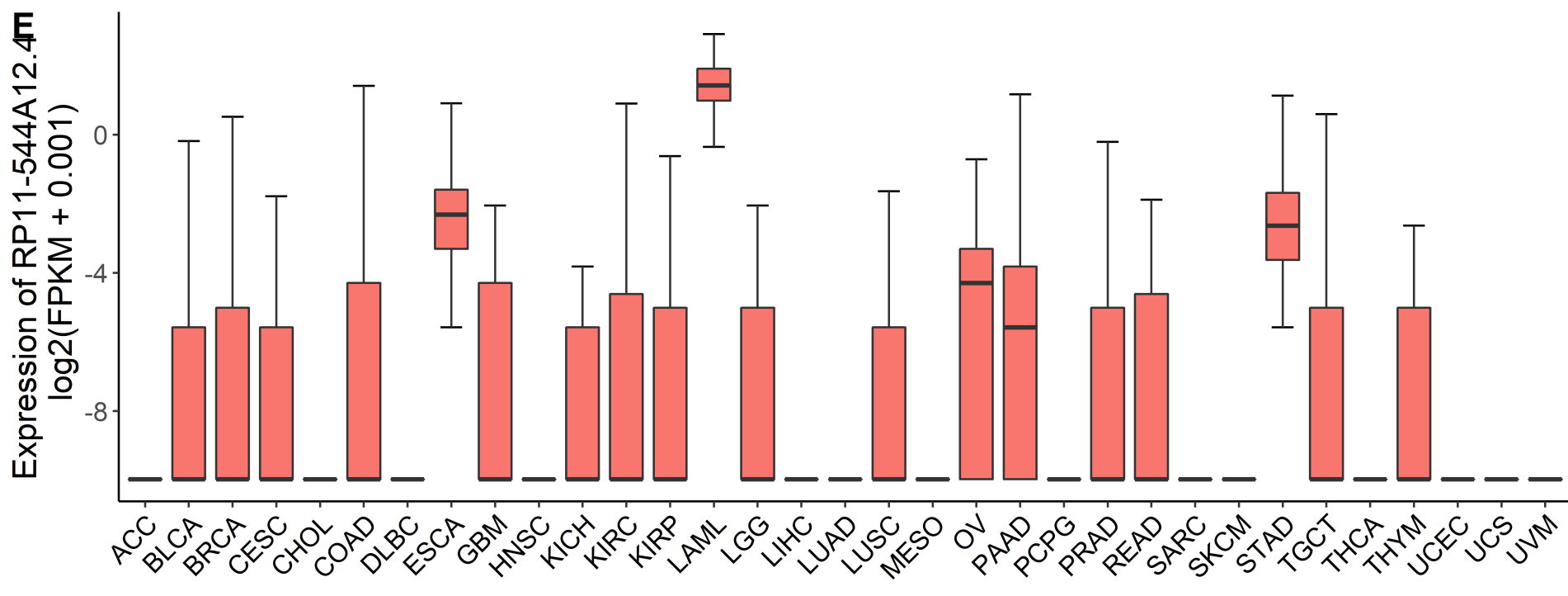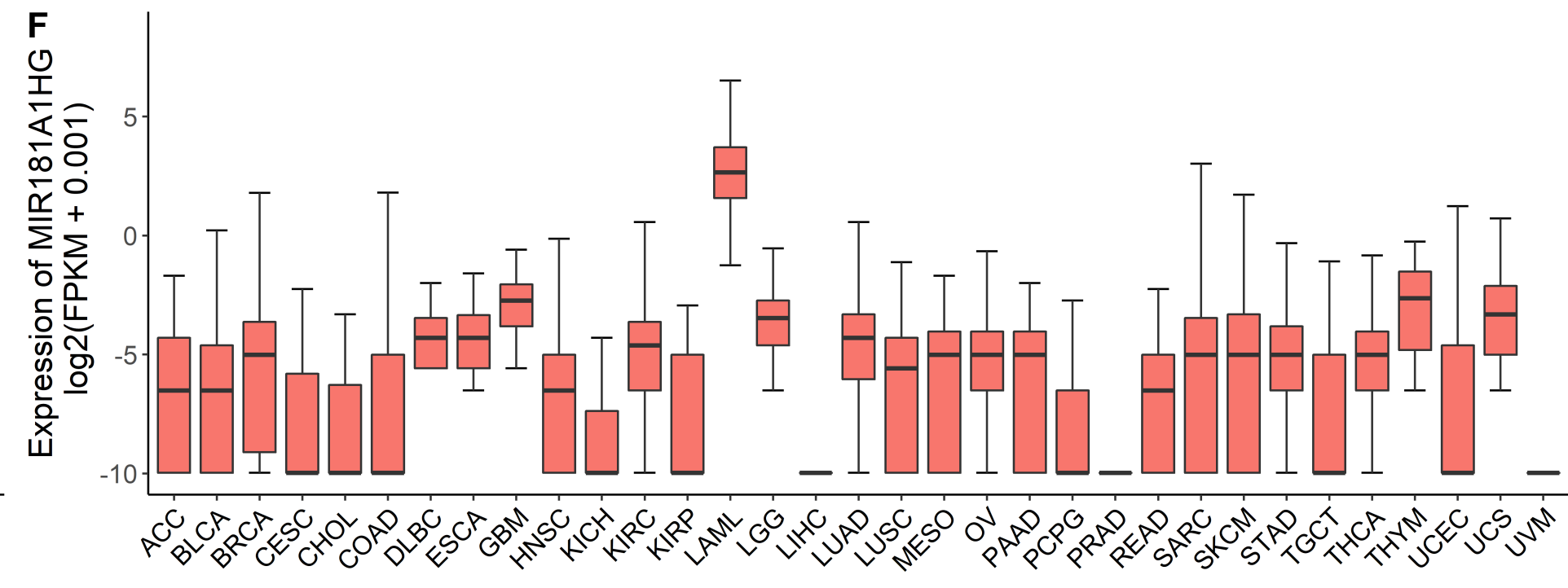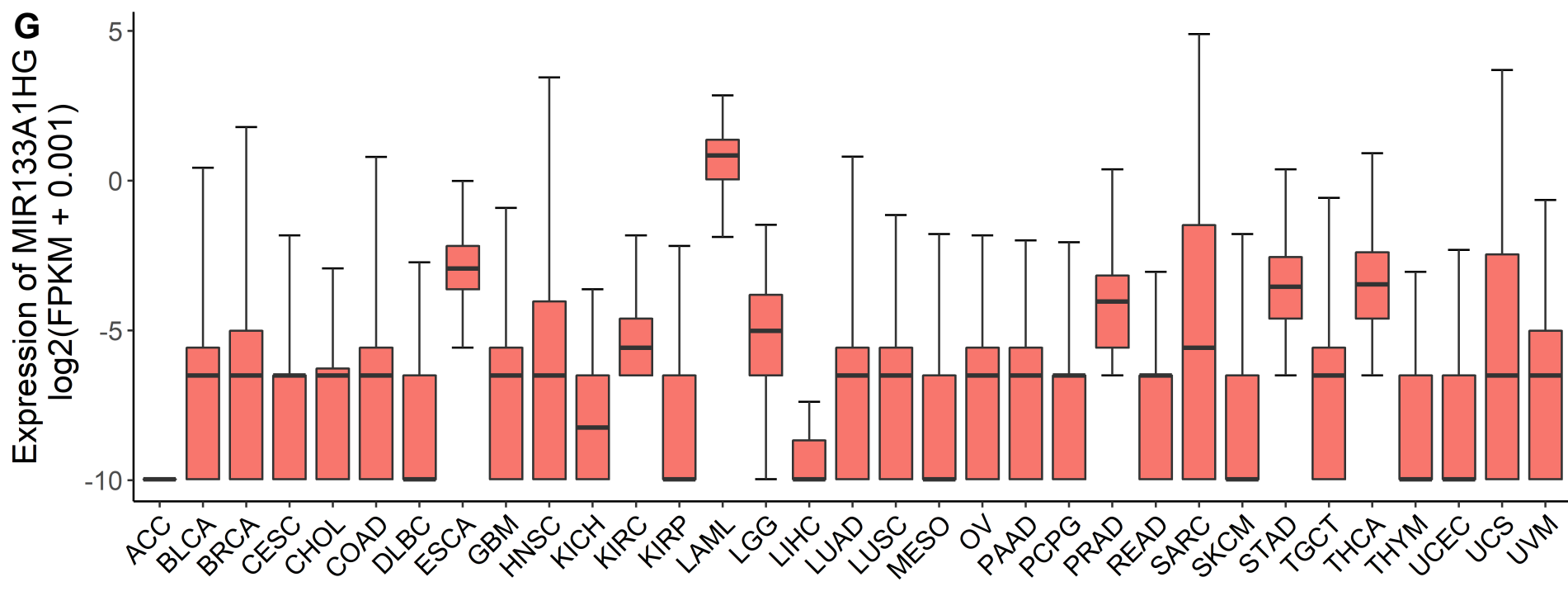

Supplement: Supplementary file 3 [file DataSheet4.pdf]

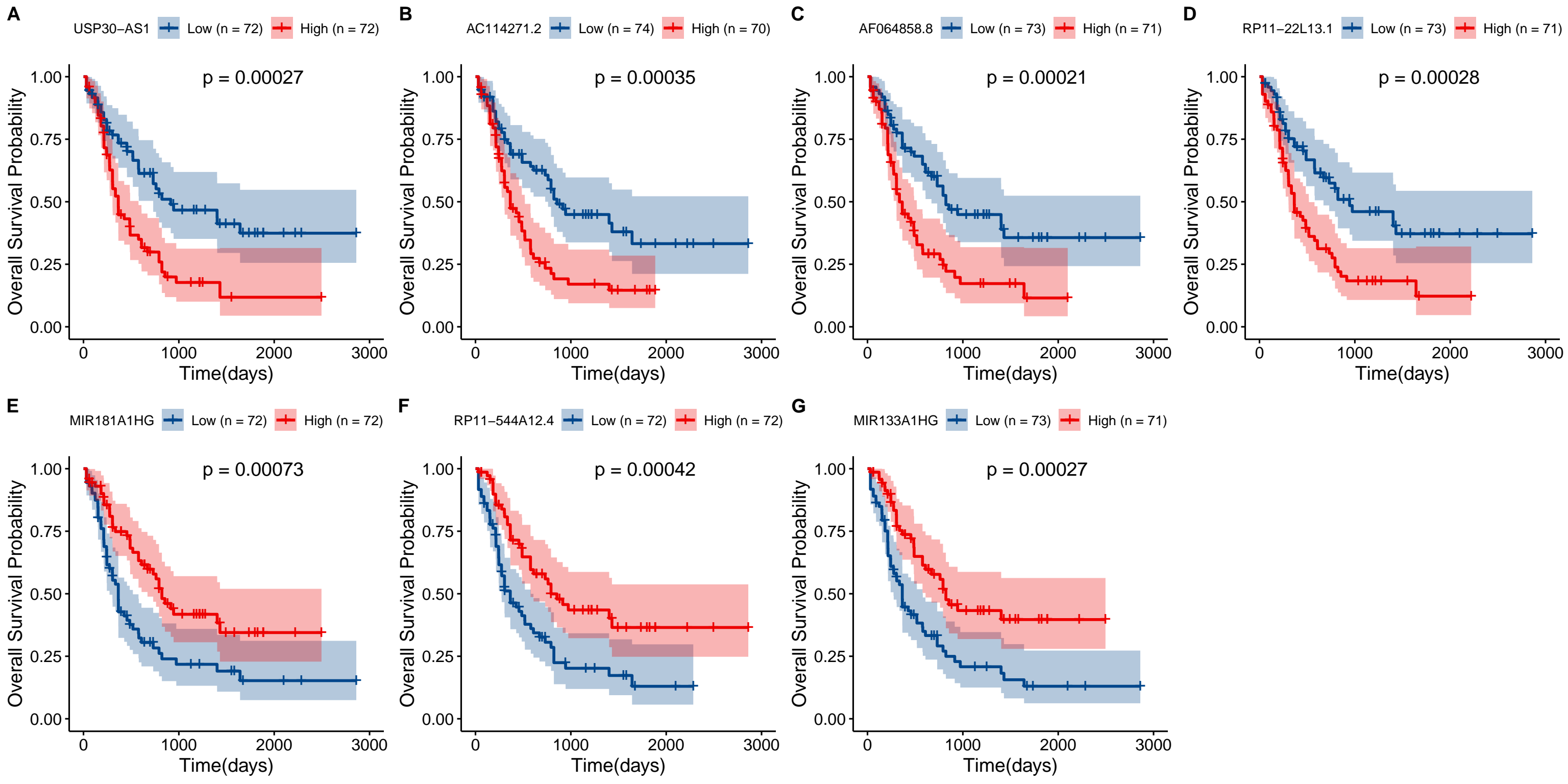

Supplement: Supplementary file 6 [file DataSheet1.pdf]

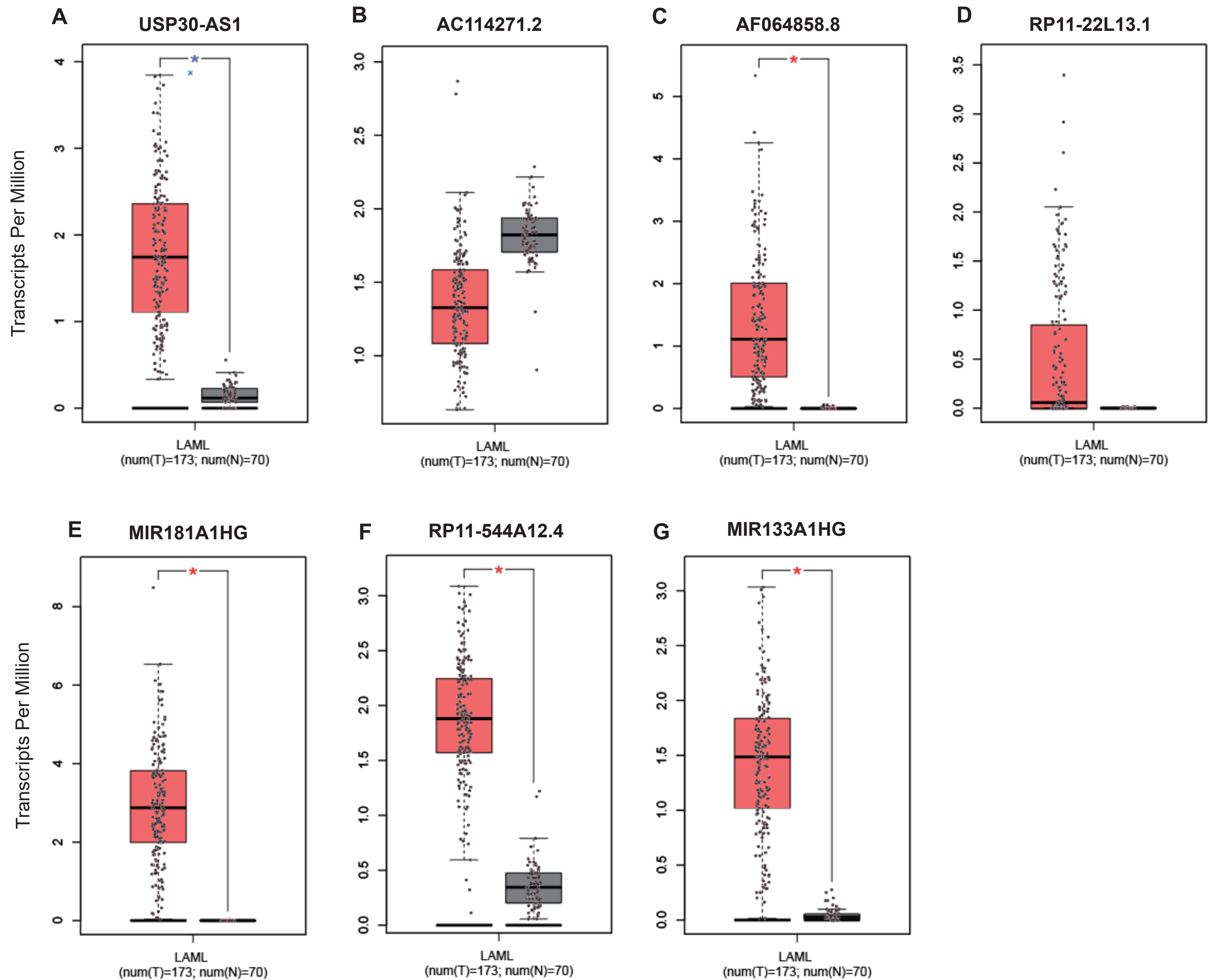

Supplement: Supplementary file 7 [file DataSheet5.pdf]
