# Supplementary material for: Identification of m6A-Related lncRNAs Associated With Prognoses and Immune Responses in Acute Myeloid Leukemia
Source: Front Cell Dev Biol. 2021 Nov 16;9:770451. doi: 10.3389/fcell.2021.770451 (PMC8637120; doi:10.3389/fcell.2021.770451)
Supplement: Supplementary file 4 [file DataSheet6.pdf]

Table S1 The clinical characteristics for AML cases in TCGA

Characteristic	High, N = 72[1]	Low, N = 72[1]	p-value[2]
Age	60 (50, 67)	53 (42, 62)	0.016
Sex			0.4
Female	31 (43%)	36 (50%)	
Male	41 (57%)	36 (50%)	
FLT3			0.3
Negative	47 (69%)	53 (77%)	
Positive	21 (31%)	16 (23%)	
NPM1			0.4
Negative	51 (73%)	56 (79%)	
Positive	19 (27%)	15 (21%)	
RAS			0.5
Negative	64 (91%)	67 (94%)	
Positive	6 (8.6%)	4 (5.6%)	
IDH1			0.12
Negative	61 (87%)	54 (77%)	
Positive	9 (13%)	16 (23%)	

[1]Median (IQR); n (%)

[2]Wilcoxon rank sum test; Pearson's Chi-squared test
