# Supplementary material for: Identification of m6A-Related lncRNAs Associated With Prognoses and Immune Responses in Acute Myeloid Leukemia
Source: Front Cell Dev Biol. 2021 Nov 16;9:770451. doi: 10.3389/fcell.2021.770451 (PMC8637120; doi:10.3389/fcell.2021.770451)
Supplement: Supplementary file 5 [file DataSheet3.pdf]

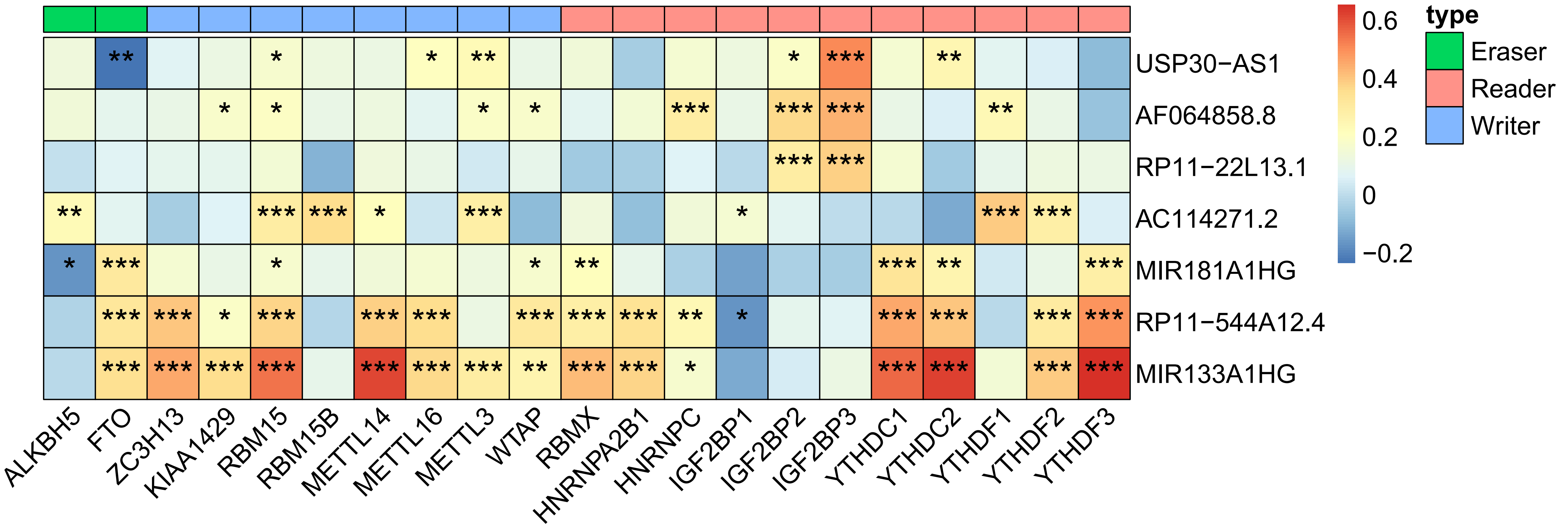

type
Eraser
Reader
Writer
0.6
0.4
0.2
0
−0.2
USP30−AS1
AF064858.8
RP11−22L13.1
AC114271.2
MIR181A1HG
RP11−544A12.4
MIR133A1HG
ALKBH5
FTO
ZC3H13
KIAA1429
RBM15
RBM15B
METTL14
METTL16
METTL3
WTAP
RBMX
HNRNPA2B1
HNRNPC
IGF2BP1
IGF2BP2
IGF2BP3
YTHDC1
YTHDC2
YTHDF1
YTHDF2
YTHDF3
